# Tethering in RNA: An RNA-Binding Fragment Discovery Tool

**DOI:** 10.3390/molecules20034148

**Published:** 2015-03-04

**Authors:** Kiet Tran, Michelle R. Arkin, Peter A. Beal

**Affiliations:** 1 Department of Chemistry, University of California, One Shields Ave, Davis, CA 95616, USA; E-Mail: ktutran@ucdavis.edu; 2 Small Molecule Discovery Center, Department of Pharmaceutical Chemistry, University of California, San Francisco, CA 94158, USA; E-Mail: Michelle.Arkin@ucsf.edu

**Keywords:** Tethering, miRNA, nucleoside analog

## Abstract

Tethering has been extensively used to study small molecule interactions with proteins through reversible disulfide bond forming reactions to cysteine residues. We describe the adaptation of Tethering to the study of small molecule binding to RNA using a thiol-containing adenosine analog (A_SH_). Among 30 disulfide-containing small molecules screened for efficient Tethering to A_SH_-bearing RNAs derived from pre-miR21, a benzotriazole-containing compound showed prominent adduct formation and selectivity for one of the RNAs tested. The results of this screen demonstrate the viability of using thiol-modified nucleic acids to discover molecules with binding affinity and specificity for the purpose of therapeutic compound lead discovery.

## 1. Introduction

The structural complexity and functional diversity of cellular RNAs make them attractive targets for high affinity small molecule ligands [1,2]. However, a significant challenge in the development of RNA-binding molecules is the difficulty in optimizing lead compounds from initial screening hits. This is due, at least in part, to difficulties in characterizing structures of RNA targets bound to weakly interacting lead compounds [3,4]. Here we describe the application of the Tethering screening method to the discovery of RNA-interacting fragments. In the Tethering method, lead molecules are covalently bound to the target such that future structural studies are not complicated by weak fragment interactions or multiple binding modes. Tethering has been successfully used in the generation of potent enzyme and protein-protein interaction inhibitors, but has yet to be applied to RNA [5]. For protein targets, Tethering involves the introduction of individual cysteine mutations surrounding the site of interest followed by screening against libraries of disulfide-containing fragments [6]. The disulfide exchange reactions allow identification of ligands since the reversible protein-fragment interactions increase the local concentration of the small molecules at the targeted site. Fragments having affinity for the protein, apart from the disulfide bond, are efficiently conjugated at equilibrium. Hits are then detected by mass spectrometry of the mixture. Fragment-target covalent conjugates can then be structurally characterized and guide the improvement of affinity and conversion to noncovalent inhibitors by subsequent modification [7]. To adapt Tethering to the discovery of RNA-binding ligands, one must introduce a thiol near the site of interest in the RNA. This can be achieved using thiol-bearing nucleoside analogs. In this initial report on Tethering for RNA, we endeavored to discover fragments that bind to a biosynthetic precursor to a biologically important miRNA, miR21. 

MicroRNAs (miRNAs) are endogenously encoded ~23 nt RNA molecules that control gene expression at the post-transcriptional level [8]. Hundreds of human miRNAs are known with each miRNA regulating multiple targets. Furthermore, altered miRNA expression correlates with human disease states, including cancer [9]. Indeed, overexpression of certain miRNAs is known to cause malignant phenotypes and these miRNAs are targets for the development of cancer therapeutics. Among these is miR-21, whose expression is dramatically upregulated in many solid tumors [10]. MiRNAs are biosynthesized from hairpin precursors via the action of two duplex-specific endonucleases, Drosha and Dicer [11]. Given this biosynthetic pathway, small molecules that bind to functionally significant sites on miRNA precursors should block their conversion to the mature miRNA. Several recent reports highlight this attractive approach that will undoubtedly benefit from the discovery of new RNA-targeting compounds that have the requisite properties to be useful starting points for drug discovery efforts [12,13,14,15]. Here we use Tethering to identify molecular fragments with affinity for pre-miR-21 near the Dicer cleavage sites. We envision such fragments providing starting structures for the development of inhibitors of the biosynthesis of miR-21. 

## 2. Results and Discussion

### 2.1. Synthesis of N^6^-(2-(triphenylmethylthio)ethyl)adenosine Phosphoramidite and Reactivity of Modified RNAs

**Figure 1:**
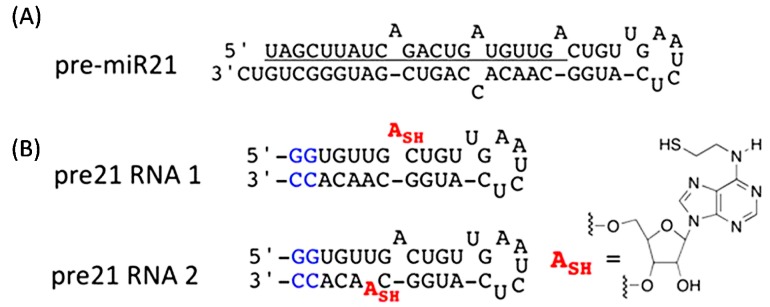
Pre-miR21 sequence-based RNAs bearing the thiol tether. (**A**) Sequence and secondary structure for pre-miR21. Underlined sequence is mature miR21; (**B**) Corresponding truncated pre21 RNA1 and pre21 RNA2 31-nucleotide oligomers (left) containing the adenosine modification A_SH_ (right). Two non-native G-C base pairs (blue) have been added to the duplex end for stability.

**Scheme 1:**
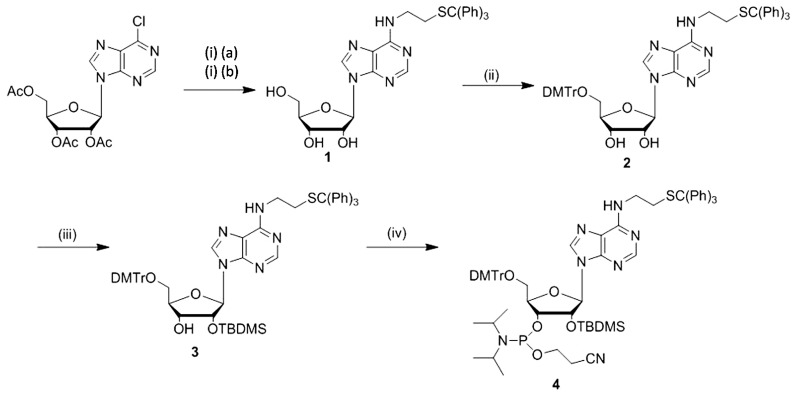
Synthesis of N^6^-(2-(triphenylmethylthio)ethyl)-adenosine phosphoramidite. *Reagents and conditions*: (i) (a) S-trityl-2-aminoethanethiol, DMF (b) NH_3_(sat) in MeOH, 72%; (ii) DMTr-Cl, DMAP, pyridine, 88%; (iii) TBDMS-Cl, N(Et)_3_, DMAP, THF, 43%; (iv) 2-cyanoethyl-(*N,N*-diisopropylamino)chlorophosphite, DIPEA, CH_2_Cl_2_, 82%.

To introduce a thiol into RNA screening targets, we synthesized a derivative of adenosine (A_SH_) that bears an ethane thiol substituent on N6 (Figure 1). This modification does not interfere with Watson-Crick hydrogen bonding and directs the reactive thiol into the major groove of an RNA duplex. Verdine and colleagues described the synthesis of RNA bearing this modification using a convertible nucleoside approach [16]. We chose to synthesize the S-trityl protected phosphoramidite, which had not been previously reported (Scheme 1). While many methods could be used to introduce a protected thiol moiety into a ribonucleoside [17,18], our previous work showed that a trityl-protected thiol group in RNA could be easily deprotected with AgNO_3(aq)_ [19].

First, the tri-*O*-acetyl protected 6-chloropurine nucleoside was allowed to react with S-trityl protected 2-aminoethanethiol in an S_N_Ar reaction to give the *N*^6^-alkylated adenosine analog. Removal of the acyl protecting groups with methanolic ammonia yielded free nucleoside **1**. Protection of the 5' and 2' hydroxyls of the ribose moiety was accomplished with DMTr-Cl and TBDMS-Cl to yield compounds **2** and **3**, respectively. Finally, the 3'-OH was allowed to react with β-cyanoethyl-(*N,N*-diisopropyl)chlorophosphite to give the corresponding phosphoramidite **4** which was then used to incorporate the nucleoside analog via solid phase synthesis into two different RNAs mimicking the hairpin loop of pre-miR21 (pre21 RNA 1 and pre21 RNA 2, Figure 1). These two RNAs differ in the positioning of the reactive adenosine analog near the Dicer cleavage sites on opposite sides of the duplex. The modified RNAs still containing S-trityl protecting groups were purified via denaturing polyacrylamide gel electrophoresis. 

**Figure 2:**
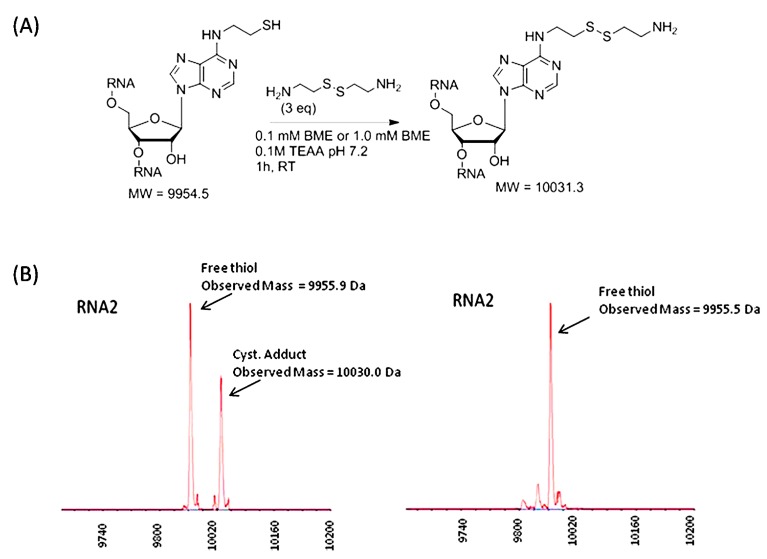
Testing the reactivity of a thiol-modified RNA. (**A**) Reaction of pre21 RNA 2 free-thiol with cystamine; (**B**) ESI-MS spectra of the disulfide conjugation reaction in 0.1 mM BME (left) and 1.0 mM BME (right).

Removal of the trityl protecting group on the sulfur was necessary before subjecting the RNA to Tethering. This was accomplished using aqueous silver nitrate giving the RNA free thiol. Deprotected pre21 RNA 2 (free thiol) and cystamine were then used to determine the appropriate conditions needed for the disulfide screen (Figure 2). Detection of reaction products was performed with ESI-MS and the relative abundances of species in a sample were determined from the areas of the peaks. The result clearly showed that the thiol-containing oligonucleotide can form a disulfide linkage with cystamine under the experimental conditions in Figure 2A, to give a product peak observed at 10,030 amu in the mass spectrum shown. The percent abundance of the cysteamine adduct was 33% in the presence of 0.1 mM β-mercaptoethanol (BME). The concentration of BME to be used in the screen should be high enough such that the majority of the small molecules would not conjugate efficiently with the RNA. Another experiment was performed using pre21 RNA 2 to determine the concentration of BME necessary to completely inhibit cystamine conjugation. The results in Figure 2B showed that no adduct could be detected at a concentration of 1.0 mM BME. Therefore, the screen was performed at a concentration of 0.5 mM BME. 

### 2.2. RNA Tethering Screen with pre21 RNA 1 and pre21 RNA 2

**Figure 3:**
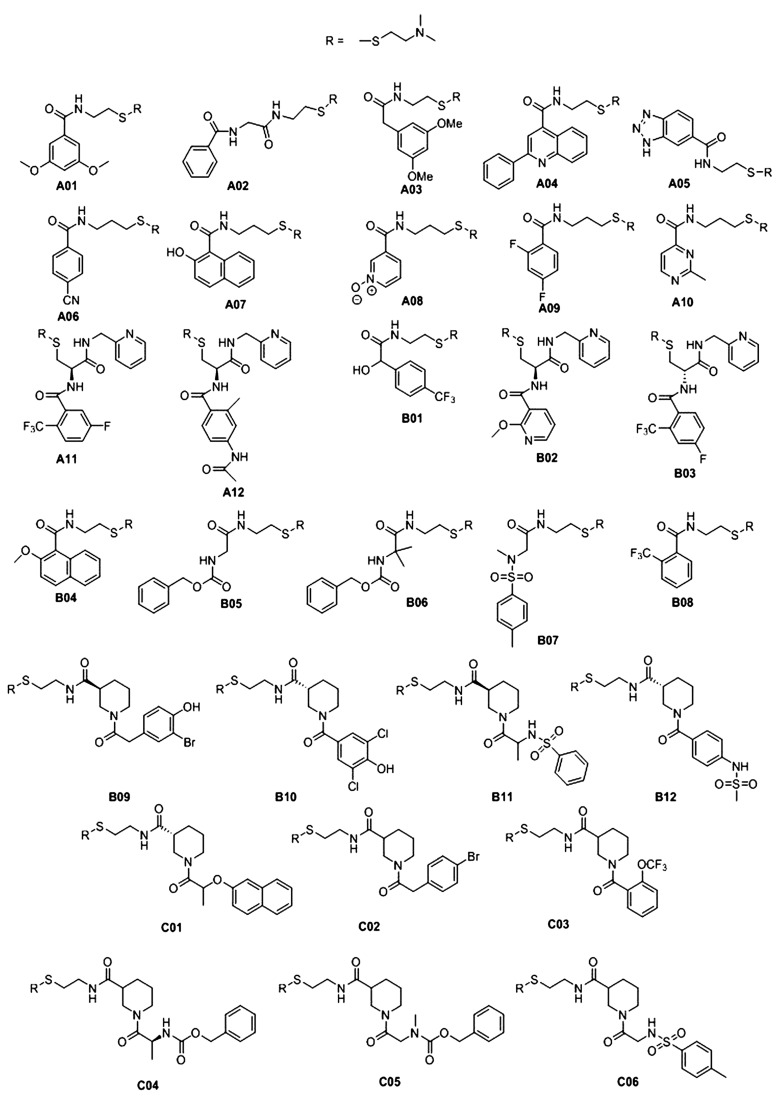
Structures of the thirty disulfide-containing small molecules used in RNA Tethering screen.

**Figure 4:**
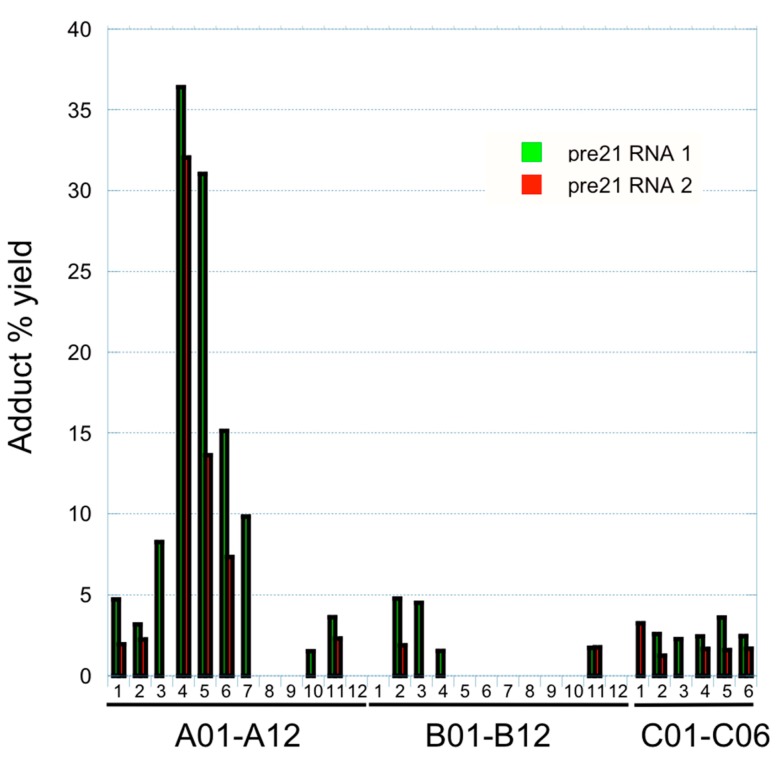
Results of an RNA Tethering screen with disulfide containing small molecules and pre21 RNA 1 and pre21 RNA 2. Plotted is the % yield of the target adduct *vs*. compound used (see Figure 3 for structures of disulfides and Experimental Section for reaction conditions.) No bar indicates no product detected in that reaction by ESI-MS.

**Figure 5:**
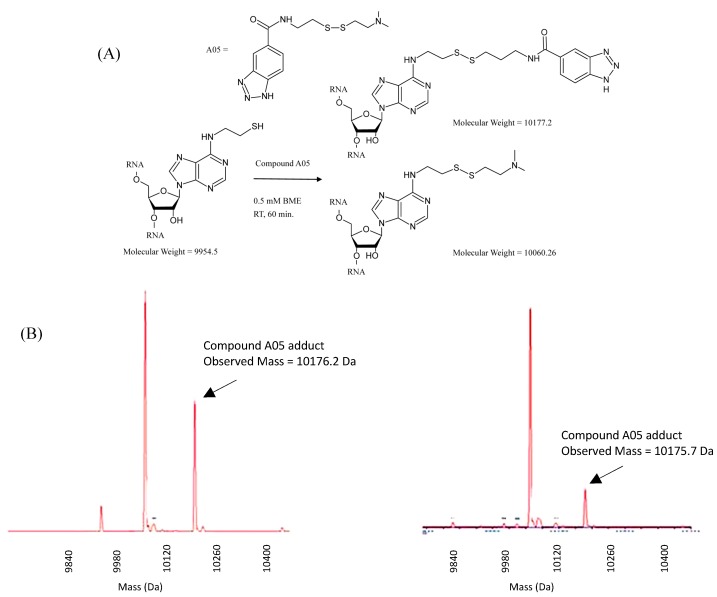
Selectivity was observed during the screen. (**A**) RNA Tethering reaction of pre21 RNA 1 and pre21 RNA 2 with compound A05; (**B**) Corresponding ESI-MS spectra for these reactions (pre21 RNA 1 (left) and pre21 RNA 2 (right)).

A small library of thirty disulfide-containing small molecules, all containing an *N,N-*dimethylethylamino (*N,N*-DMA) moiety for solubility purposes, was obtained from the Small Molecule Discovery Center, UCSF for the Tethering screen (Figure 3) [20]. Each compound at 0.12 mM concentration was allowed to react with 20 µM of thiol-bearing RNA in 0.1 M TEAA buffer (pH 7.2) for 1 h at room temperature. The resulting sixty reaction mixtures were then analyzed by ESI-MS and percent relative abundance of the target adduct was used to assess small molecule binding efficiency. The results of the screen are shown in Figure 4, Supplementary Table S1 and Supplementary Table S2. In addition, the ESI-MS data for an example screening reaction is shown in Figure 5 with compound A05. The majority of the compounds in the library showed minimal to no target adduct formation (Figure 4). This was expected considering the concentration of reducing agent used. However, compounds A04 and A05 gave >30% target adduct formation for pre21 RNA 1 under the reducing conditions of the Tethering screen suggesting a binding interaction between the RNAs and these compounds. For compound A04, efficient conjugation was not specific to pre21 RNA 1 as pre21 RNA 2 also gave a high conjugate yield. This is perhaps not surprising considering the structure of A04 (Figure 3). 2-Phenylquinolines are known RNA ligands that bind by an intercalation mechanism [21]. Such a binding mode is possible at multiple sites on these RNAs consistent with efficient reaction independent of Tethering site. While this result is not desirable for the development of selective ligands, it does provide confirmation of the validity of this screening method as a means of discovering RNA-interacting ligands. In contrast to A04, compound A05 gave a high conjugate yield (31%) with pre21 RNA 1 and a lower yield (13%) with pre21 RNA 2 suggesting a selective binding site near the bulged adenosine for the benzotriazole appendage of A05 (Figure 4 and Figure 5). Furthermore, when the reaction with A05 was carried out with an unrelated thiol-modified 16 bp duplex, a low conjugate yield was observed (<5%) while compound A04 produced conjugate with this RNA in 38% yield (Supplementary Information). Interestingly, the benzotriazole structure of A05 can be considered a base analog with the potential to stack and hydrogen bond like natural nucleobases. Indeed, tetrabromobenzotriazole is a known inhibitor of nucleotide-dependent enzymes where the inhibitor interacts with nucleotide-binding sites [22]. A recent NMR study of the structure of this part of the pre-miR21 RNA indicates the bulged A is extrahelical and partially associated with a groove of the adjacent duplex [23]. It is possible that the benzotriazole of A05 contacts the RNA groove at this location. However, at this point, we cannot rule out the possibility that our covalent modification strategy altered the RNA structure, placing the bulged A in a different position. Nevertheless, if the binding site for the tethered benzotriazole is present in the native RNA, it can provide a useful starting point for the development of selective pre-miR21 ligands. Additional structural studies will be necessary to fully define this binding site. 

## 3. Experimental Section 

### 3.1. General Procedures

All reagents and solvents were purchased from Sigma/Aldrich or Fischer Scientific and were used without additional purification, unless noted otherwise. Reactions using dry solvents were performed under an atmosphere of argon, in oven-dried glassware. Solvents were dried in a solvent purification system that passes solvent through two columns of dry neutral alumina. Column chromatography was conducted using silica gel (Sorbent Technologies, 60–200 mesh) and analytical TLC was performed on glass plates coated with 0.25 mm silica gel using UV for visualization. ^1^H and ^13^C magnetic resonance spectra were recorded with Varian VNMRS 600, Varian Mercury 300 spectrometers and referenced to the residual solvent peak. ^31^P magnetic resonance spectra was recorded with a Varian Mercury 300 spectrometer. The abbreviations s, t, m, brs, dd, d stand for singlet, triplet, multiplet, broad singlet, doublet of doublets and doublet. High-resolution ESI mass spectra were obtained at the University of California, Davis Mass spectrometry facility, on a Thermo Electron LTQ-Orbitrap Hybrid Mass Spectrometer.

*N^6^-[2-(triphenylmethylthio)ethyl]-9-(β-d-ribofuranosyl)adenine* (**1**):

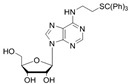



2',3',5'-Tri-O-acetyl-9-(β-D-ribofuranosyl)-6-chloropurine (100 mg, 0.218 mmol) was dissolved in 1.0 mL anhydrous DMF and treated with S-trityl-protected 2-aminoethanethiol [24] (161 mg, 2.3 equiv) in 1.0 mL DMF. After stirring for 18 h, NH_3_/MeOH (5 mL, saturated solution) was added, and stirring continued for a further 15 h. The crude was then diluted with 10 mL CH_2_Cl_2_, extracted twice with saturated NaCl, and dried over Na_2_SO_4_. The crude mixture was then concentrated under reduced pressure, absorbed onto silica gel, and purified by column chromatography (0% → 6% MeOH in CH_2_Cl_2_), yielding a pale yellow solid (85 mg, 72%). ^1^H-NMR (300 MHz, CD_3_OD) δ 8.25 (s, 1H), 8.13 (s, 1H), 7.40-7.07 (m, 15H), 5.96 (d, *J =* 6.4 Hz, 1H), 5.49 (s, 1H), 4.76 (t, *J =* 6.0 Hz, 1H), 4.33 (d, *J =* 2.6 Hz, 1H), 4.18 (s, 1H), 3.90 (d, *J =* 12.5 Hz, 1H), 3.75 (d, *J =* 12.7 Hz, 1H), 3.49 (s, 1H), 2.54 (t, *J =* 6.5 Hz, 2H), 2.39 (dd, *J =* 14.5, 5.6 Hz, 4H). ^13^C NMR (151 MHz, CDCl_3_) δ 154.53, 152.15, 144.54, 139.92, 146.79, 129.49, 127.86, 127.20, 126.68, 91.10, 87.65, 73.63, 73.64, 72.59, 67.92, 39.22, 31.67, 29.64, 25.54. ESIHRMS: calcd for C_31_H_31_N_5_O_4_S (M+H)^+^: 570.2169, obsd 570.2168. 

*N6-[2-(triphenylmethylthio)ethyl]-9-[5-O-(4,4’-dimethoxytrityl)-β-d-ribofuranosyl]adenine* (**2**):

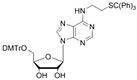



DMTrCl (229 mg, 1.1 eq) and DMAP (37 mg, 0.5 eq) were added to a solution of **1** (350 mg, 0.61 mmol) in 5 mL anhydrous pyridine. The reaction mixture was stirred at RT for 16 h. Co-evaporation with 20 mL tolune and 20 mL CH_3_CN was performed to remove pyridine and toluene, respectively. Afterwards, the mixture was diluted with CH_2_Cl_2_ (15 mL) and washed with saturated aqueous NaHCO_3_ (2 × 10 mL). The organic layer was dried (Na_2_SO_4_), concentrated under reduced pressure and purified by column chromatography (1% Et_3_N in CH_2_Cl_2_). Excess Et_3_N in column fractions were removed via azeotrope formation with CH_3_CN, yielding a white solid (467 mg, 88%). ^1^H-NMR (300 MHz, CD_2_Cl_2_) δ 8.22 (s, 1H), 8.03 (s, 1H), 7.45–7.19 (m, 24H), 6.79 (m, 4H), 6.26 (d, *J =* 5.4 Hz, 1H), 5.99 (d, *J =* 5.2 Hz, 1H), 4.73 (t, *J =* 4.8 Hz, 1H), 4.40 (d, *J =* 4.5 Hz 1H), 3.75 (s, 6H), 3.47–3.29 (m, 4H), 2.62–2.51 (m, 2H), 2.31 (t, *J =* 6.4 Hz, 2H). 13C-NMR (75 MHz, CD_2_Cl_2_) δ 158.57, 152.29, 144,72, 144.59, 138.04, 135.54, 135.38, 129.91, 129.87, 129.49, 129.47, 127,90, 127.83, 127.75, 126.74, 126.66, 90.84, 86.32, 86.11, 76.02, 72.61, 66.76, 63.55, 55.14, 38.06, 31.85, 29.67, 22.92. ESIHRMS: calcd for C_52_H_49_N_5_O_6_S (M+H)^+^: 872.3476, obsd: 872.3473. 

*N^6^-[2-(triphenylmethylthio)ethyl]-9-[2-O-(tert-butyldimethylsilyl)-5-O-(4,4’-dimethoxytrityl)-β- d-ribofuranosyl]adenine* (**3**):

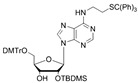



In a flame-dried 25 mL round-bottom flask, TBDMSCl (93 mg, 1.2 eq), freshly distilled triethylamine (145 uL, 2.0 eq) and DMAP (32 mg, 0.5 eq) were added to a solution 5'-*O*-DMTr protected derivative **2** (450 mg, 0.516 mmol, 1 eq) in 1 mL anhydrous THF. The reaction mixture was stirred at RT for 24 h. Upon completion of the reaction, the mixture was diluted with EtOAc (10 mL), filtered out white precipitate, and washed with 5% aqueous NaHCO_3_ (2 × 10 mL). The organic layer was dried (Na_2_SO_4_) and concentrated under reduced pressure. The crude reaction mixture was purified by column chromatography (15% → 30% (CH_3_)_2_CO in CH_2_Cl_2_) to afford a white foam (152 mg, 43%). ^1^H-NMR (300 MHz, CDCl_3_) δ 8.30 (s, 1H), 8.06 (s, 1H), 7.59–7.32 (m, 24H), 6.94 (d, *J =* 8.8 Hz, 4H), 6.09 (d, *J =* 5.0 Hz, 1H), 5.97 (s, 1H), 5.10 (s, 1H), 4.42 (d, *J =* 4.2 Hz, 1H), 4.32 (d, *J =* 4.2 Hz, 1H), 3.89 (s, 6H), 3.54 (m, 4H), 2.78 (d, *J =* 4.6 Hz, 1H), 2.68 (t, *J =* 6.6 Hz, 2H), 2.13 (s, 1H), 0.97 (s, 9H), 0.12 (s, 3H), 0.00 (s, 3H). 13C-NMR (75 MHz, CD_2_Cl_2_) δ 158.59, 144.80, 144.72, 138.73, 135.65, 135.62, 130.01, 130.00, 129.47, 128.01, 127.81, 127.77, 126.75, 126.61, 113.02, 88.37, 86.40, 83.81, 75.26, 71.35, 63.40, 55.12, 29.62, 25.29, 17.73. ESIHRMS: calcd for C_58_H_63_N_5_O_6_SSi (M + H)^+^: 986.4341, obsd: 986.4370. 

*N^6^-[2-(triphenylmethylthio)ethyl]-9-[2-O-(tert-butyldimethylsilyl)-5-O-(4,4’-dimethoxytrityl)-β- d-ribofuranosyl]adenine 3'-(2-Cyanoethyl)-N,N-diisopropylphosphoramidite* (**4**)*:*

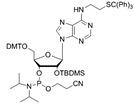



Dry diisopropylethylamine (70 uL, 6 eq) and 2-cyanoethyl-(N,N-diisopropylamino)chlorophosphite (29 uL, 2 eq) were added to a solution of **3** (74 mg, 0.075 mmol, 1 eq) in 1.0 mL dry DCM. The reaction mixture was stirred for 1 h. Upon completion of the reaction, the mixture was diluted with EtOAc (5 mL) and washed with 5% (w/v) aqueous NaHCO_3_ (2 × 5 mL). The organic layer was dried (Na_2_SO_4_) and concentrated under reduced pressure. Purification by column chromatography (30%–40% EtOAc in Hexane) yielded a white foam (62 mg, 82%). ^31^P-NMR (121 MHz, CD_2_Cl_2_) δ 151.68, 150.24. ESIHRMS: calcd for C_67_H_80_N_7_O_7_PSSi (M+H)^+^: 1186.5420, obsd: 1186.5474. 

### 3.2. RNA Synthesis, Purification and S-Tr Deprotection

RNA oligonucleotides were synthesized on an ABI 394 synthesizer (DNA/Peptide Core Facility, University of Utah) at 200 nmol and 1 umol scale using 5'-DMTr, 2'-OTBDMS protected β-cyanoethyl phosphoramidites. Conditions for synthesis, cleavage and standard deprotection are identical to those previously described [25]. Crude RNA synthesis products were purified by urea-polyacrylamide gel electrophoresis (19%), and desalted with Sep-Pak cartridges, as previously described. The S-Trityl RNA was stored as a lyophilized pellet at −70 °C. Identification and purity was determined by ESI-MS. 

Prior to S-trityl deprotection [19], the RNA was allowed to fold into its native secondary structure by heating at 95 °C for 30 min. then slow cooling in the heat block until the sample has reached room temperature. To remove the S-Trityl protecting group, purified RNA (1.2 nmol) was dissolved in aqueous TEAA buffer (triethylammonium acetate, 15 uL, 0.1 M, pH 6.5) and treated with AgNO_3_ (2 μL, 1 M in H_2_O). After 30 min of gentle agitation by vortex at room temperature, DTT was added (2 μL, 1 M H_2_O), and the reaction was allowed to proceed for a further 10 min. The mixture was diluted with aqueous 0.1 M TEAA (pH 6.5) and centrifuged (16,000× *g*, 4 °C, 5 min) to separate the precipitate. The supernatant was collected. The pellet was washed 3 times with 50 uL aqueous 0.1 M TEAA, to minimize the loss of the RNA. Each wash was followed by vigorous vortex agitation. The combined supernatants (~170 μL) were loaded in to a 3000 MWCO centrifugal concentrator (Microcon-3, Millipore) and centrifuged (11,000× *g*, 4 °C, approx. 30 min). The concentrated crude sample was then washed once with 150 μL of 0.1 M TEAA and centrifuged (11,000× *g*, 4 °C, approx. 30 min). A final wash was performed with 100 μL of nuclease-free water and centrifuged (11,000× *g*, 4 °C, approx. 30 min). After the buffer exchange into water, the deprotected RNA was quantified by the absorbance of the solution at 260 nm. Yields ranged from 340 pmol (28%) to 940 pmol (76%). ESI-MS confirmed quantitative conversion to the free thiol-RNA, and the purity of the product. The deprotected RNA was stored in aliquots of water at −20 °C if not used immediately for experimentation.

STr-protected pre21 RNA 1: 5'-GGU GUU GCX UGU UGA AUC UCA UGG CAA CAC C-3'

X = A_SH_

Calcd mass (monoisotopic): 10,195.76

Obsd mass (ESI-MS): 10,193.56

Free thiol pre21 RNA 1: 5'- GGU GUU GCX UGU UGA AUC UCA UGG CAA CAC C -3'

X = A_SH_

Calc mass (monoisotopic): 9954.5

Obsd mass (ESI-MS): 9955.5

STr-protected pre21 RNA 2: 5'-GGU GUU GCA UGU UGA AUC UCA UGG CXA CAC C-3'

X = A_SH_

Calcd mass (monoisotopic): 10,195.8

Obsd mass (ESI-MS): 10,194.3

Free thiol pre21 RNA 2: 5'- GGU GUU GCX UGU UGA AUC UCA UGG CXA CAC C-3'

X = A_SH_

Calc mass (monoisotopic): 9954.5

Obsd mass (ESI-MS): 9955.1

### 3.3. Mass Spectrometry Analysis of RNAs

Mass spectra were obtained on a Thermo Electron LTQ-Orbitrap Hybrid Mass Spectrometer. Free-thiol RNAs were desalted and purified prior to conjugate formation. Conjugate reaction samples were analyzed crude. The samples were run with a mobile phase consisting of 50% MeOH/H_2_O w/0.2% triethylamine. Mass spectra were recorded in negative ionization mode. A 9 kDa DNA standard was used to tune the instrument before each run. Spectra were deconvoluted using ProMass and MassLynx software. 

### 3.4. Conjugation Reaction Procedure

Free-thiol RNA (~200 pmol) was dissolved in 8 μL of 0.1 M TEAA (triethylammonium acetate, pH 7.2). Freshly prepared β-mercaptoethanol (1 μL, 0.5 M) was then added to the solution and pipet-mixed. The disulfide compound (1 μL, 1.25 mM in H_2_O) was added to the reaction mixture immediately. The reaction was allowed to proceed for 1 h at room temperature under gentle agitation. Samples were then analyzed with ESI-MS immediately or stored at −20 °C. 

### 3.5. Calculation of Reaction Product Abundances

Abundances were calculated using the area of the peaks in the deconvoluted spectrum of each sample reaction. All peaks were accounted for in each spectrum. The noteworthy peaks in each spectrum are as follows: (1) free thiol; (2) N,N-dimethylethylamino adduct (N,N-DMA); (3) product. Given their structural similarities, these species were assumed to have equivalent ionization efficiencies. However, due to possible variations in ionization behavior, quantification of conjugates in this manner may not always reflect exact yields. At an RNA concentration of 20 µM, no RNA dimers were observed in the spectra.

% abundance = (area of species peak/sum of the areas of all peaks in spectrum) × 100

## 4. Conclusions

We have demonstrated that oligonucleotides bearing the A_SH_ nucleoside analog can effectively conjugate with disulfide-containing small molecules under varying reducing conditions. Among the library of 30 disulfide-containing compounds screened, a small molecule bearing a benzotriazole moiety displayed not only prominent yield of the adduct, but also selectivity for pre21 RNA 1 where the A_SH_ analog replaces a bulged adenosine near the Dicer cleavage site. The result suggests a mode of binding to the RNA outside of the disulfide linkage and may provide a starting point for the development of pre-miRNA21 ligands. 

## Supplementary Materials

Supplementary materials can be accessed at: http://www.mdpi.com/1420-3049/20/03/4148/s1.
