# Supplementary material for: Tethering in RNA: An RNA-Binding Fragment Discovery Tool
Source: Molecules. 2015 Mar 4;20(3):4148–61. doi: 10.3390/molecules20034148 (PMC4760646; doi:10.3390/molecules20034148)

# Supporting Information

**Table of Contents**

1. $^{1}H$ & $^{13}C$-NMR Spectra of Compounds **1–4**........ S2
2. $^{31}P$-NMR of Compound **4**........ S5
3. Small Molecule Library Structures........ S6
4. Supplementary Table S1. Pre21 RNA1 Conjugation Data........ S7
5. Supplementary Table S2. Pre21 RNA2 Conjugation Data........ S8
6. Pre21 RNA1 Conjugation Data Mass Spectra........ S9
7. Pre21 RNA2 Conjugation Data Mass Spectra......... S24
8. 16mer Conjugation Data with A04 & A05........ S39
9. Denaturing Gel Electrophoresis of RNA2 after Solid-phase Synthesis........ S41

# 1. $^{1}$H &$^{13}$C-NMR Spectra of Compounds 1–4

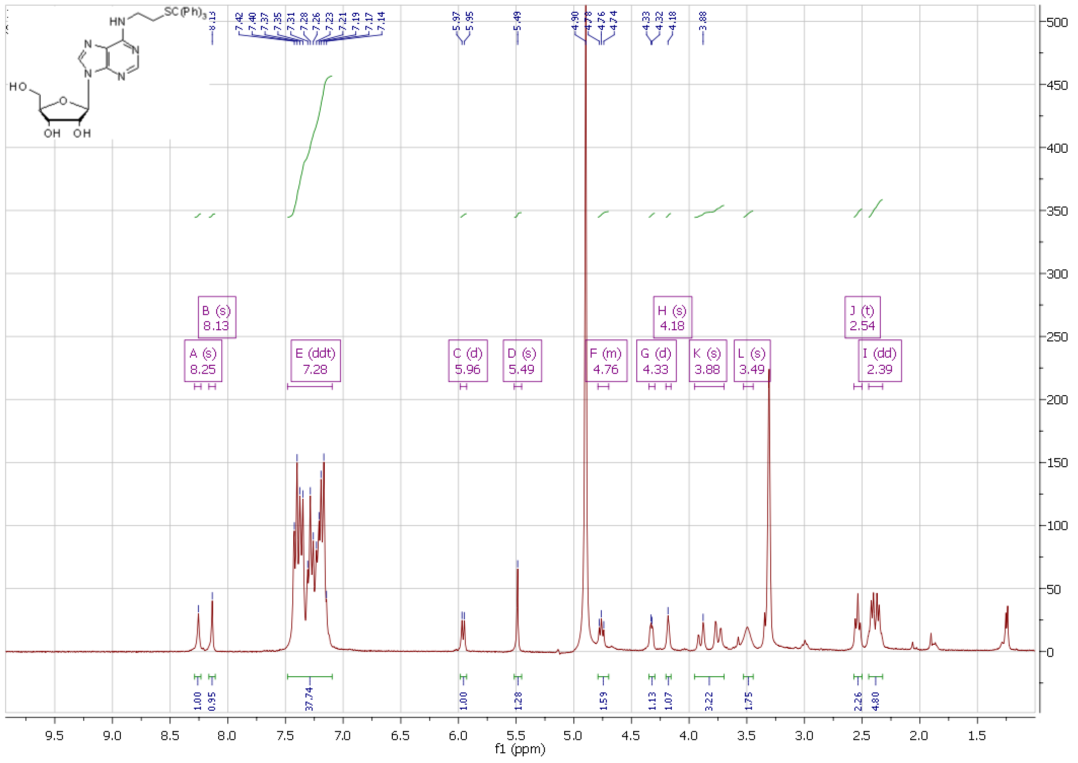


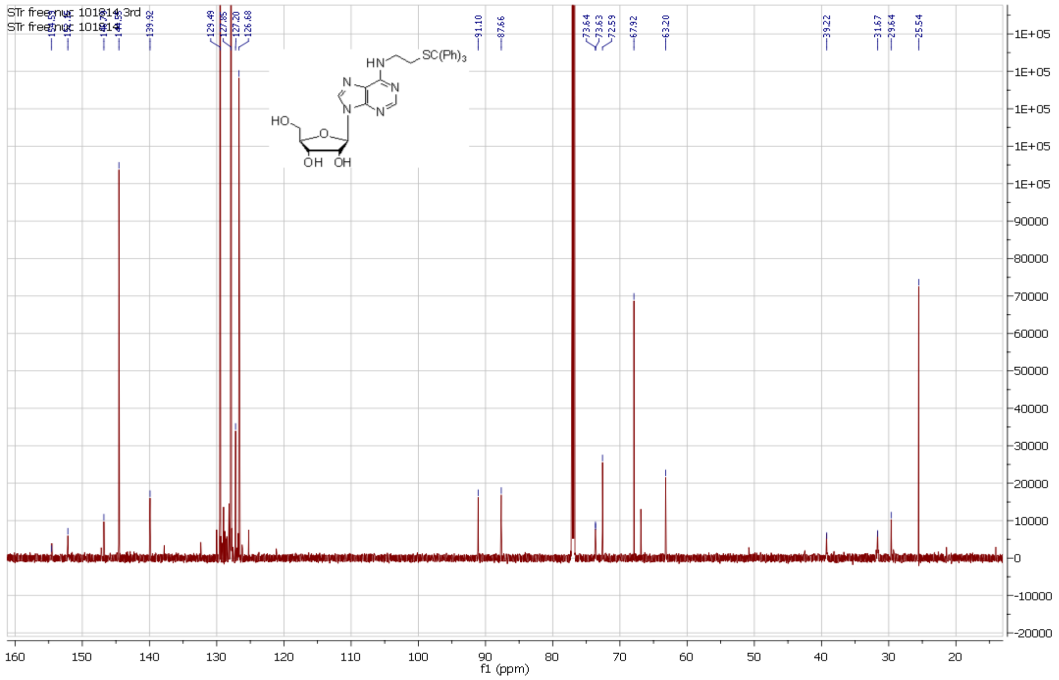


**Compound 1**

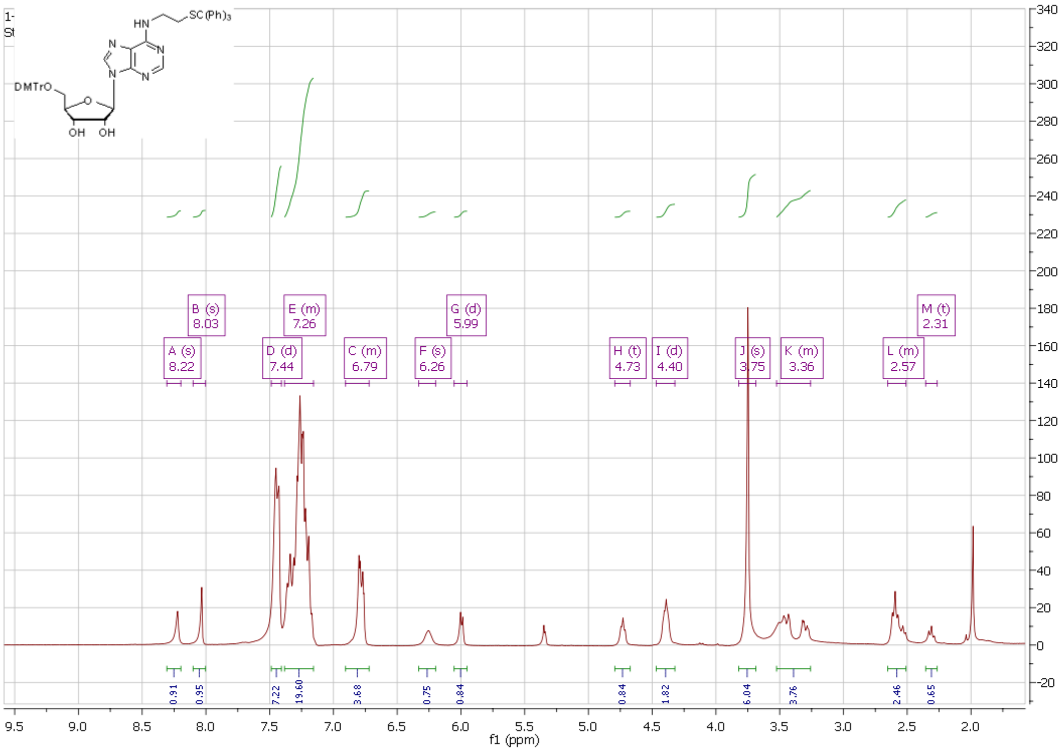

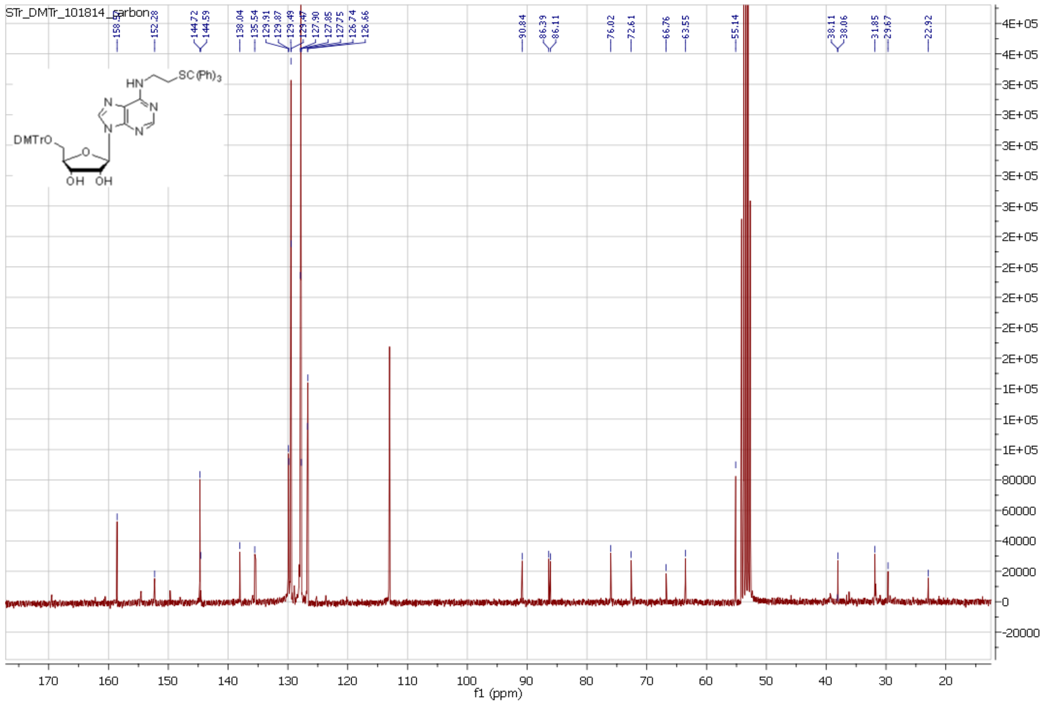

**Compound 2**

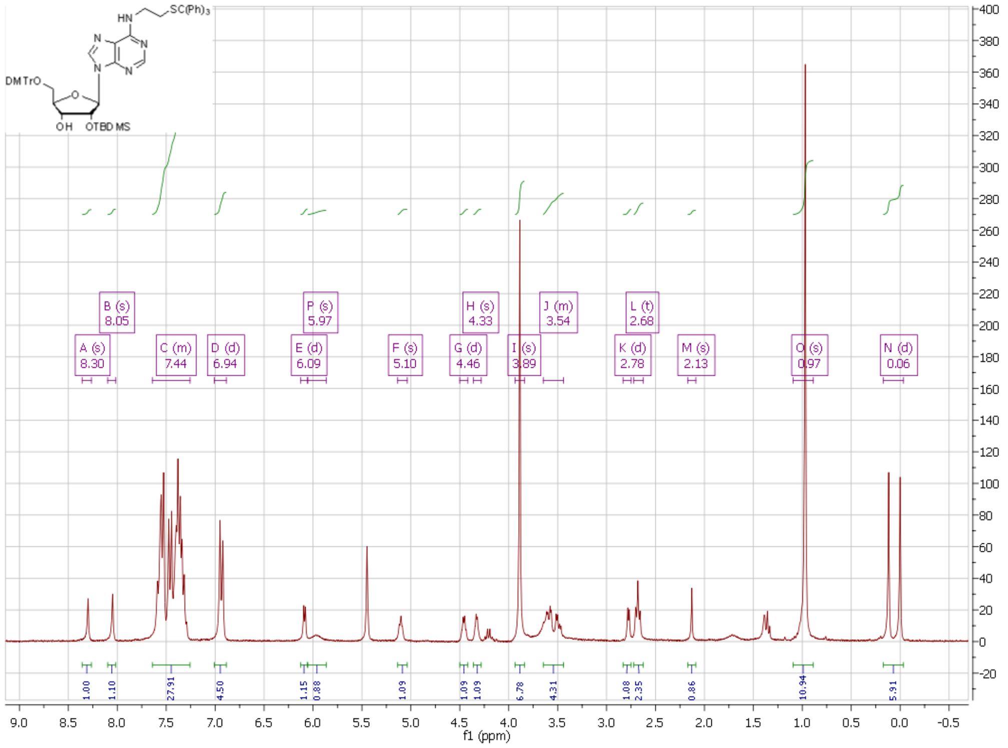

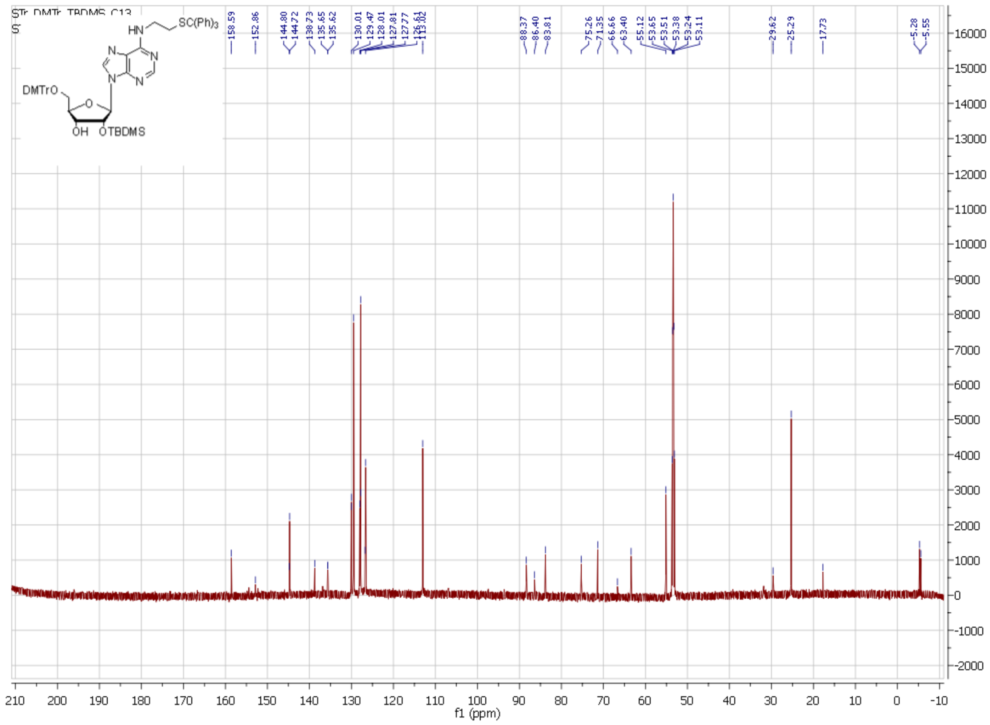

**Compound 3**

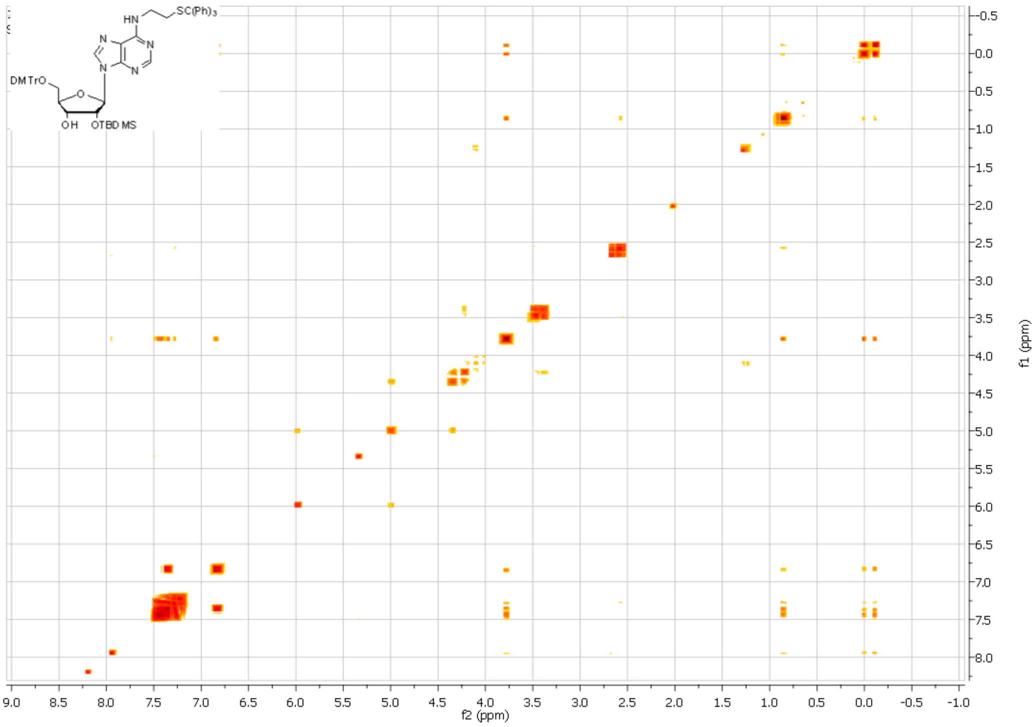


**Compound 3**

## 2. $^{31}$P-NMR of Compound 4

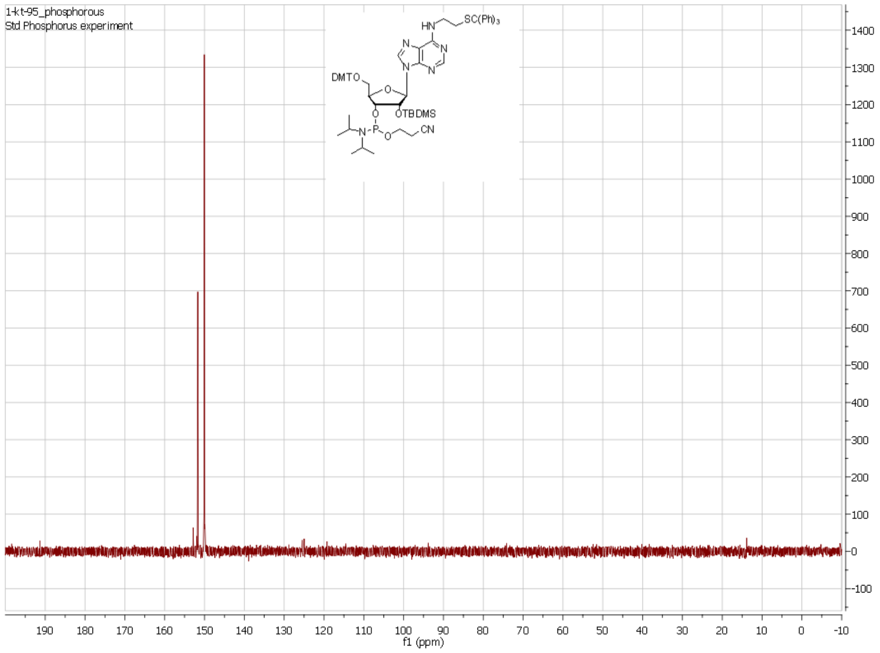


**Compound 4**

## 3. Small Molecule Library Structures

Structures for all 30 disulfide-containing small molecules used in this study.

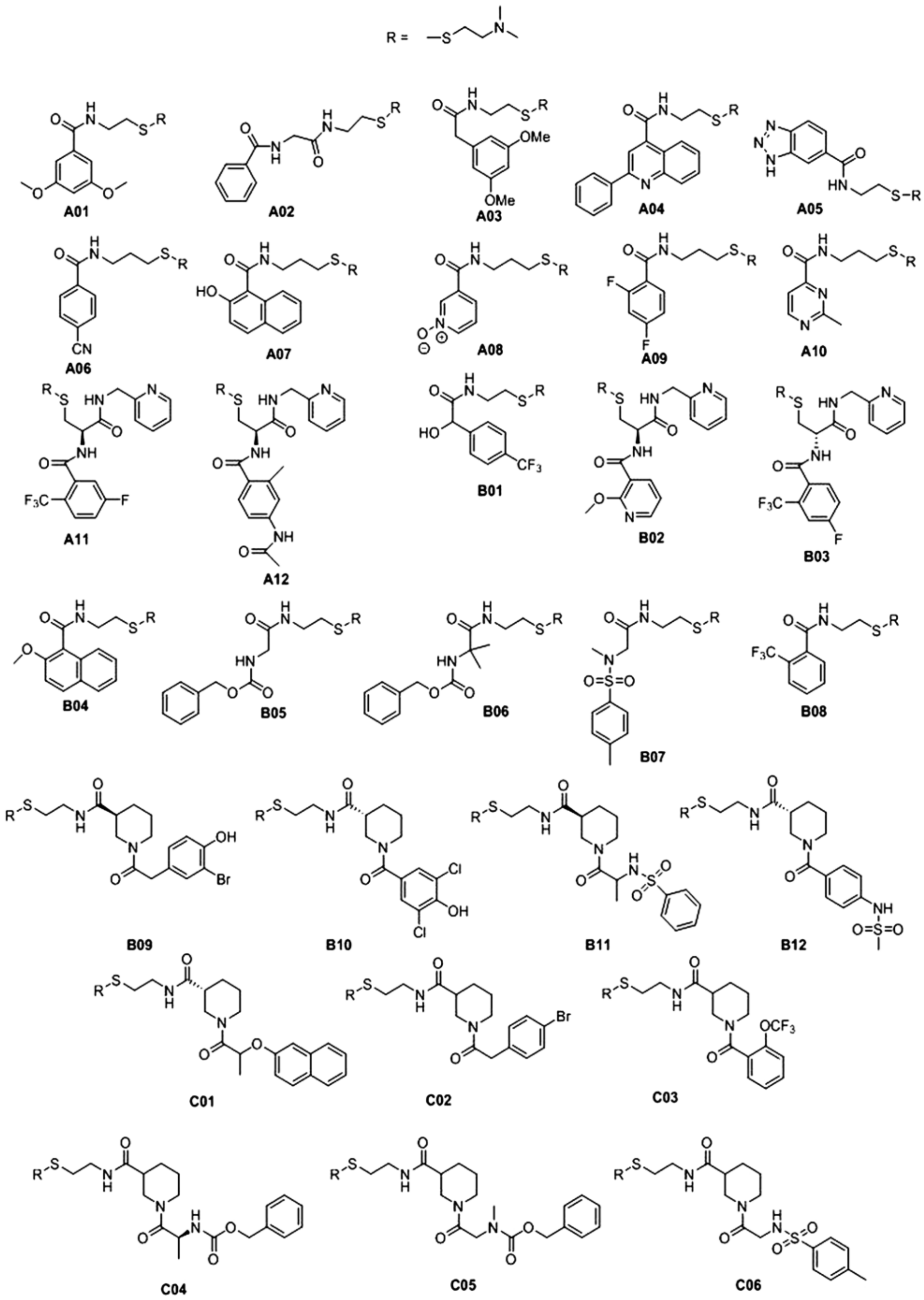

## 4. Supplementary Table S1. Pre21 RNA1 Conjugation Data

**Table S1.** Small molecule disulfide screening data for pre21 RNA1.

| % Relative Abundances | | | |
|---|---|---|---|
| **Compound** | **Thiol** | **N,N-DMA** | **Target** |
| A01 | 0 | 68.03 | 4.71 |
| A02 | 29.34 | 46.34 | 3.17 |
| A03 | 13.16 | 39.72 | 8.26 |
| A04 | 10.23 | 38.87 | 36.41 |
| A05 | 6.2 | 57.13 | 31.04 |
| A06 | 3.23 | 49.22 | 5.04 |
| A07 | 15.62 | 19.99 | 9.82 |
| A08 | 8.82 | 51.06 | 0 |
| A09 | 69.8 | 1.81 | 0 |
| A10 | 78.07 | 1.94 | 1.52 |
| A11 | 80.32 | 2.63 | 3.62 |
| A12 | 59.8 | 17.41 | 0 |
| B01 | 31.4 | 42.7 | 0:00 |
| B02 | 71.79 | 10.63 | 4.76 |
| B03 | 66.56 | 11.43 | 4.51 |
| B04 | 83.94 | 3.31 | 1.53 |
| B05 | 83.1 | 1.4 | 0 |
| B06 | 82.2 | 0 | 0 |
| B07 | 82.17 | 1.31 | 0 |
| B08 | 84.8 | 1.92 | 0 |
| B09 | 86.18 | 0 | 0 |
| B10 | 88.3 | 1.33 | 0 |
| B11 | 83.86 | 0 | 1.73 |
| B12 | 87.32 | 1.31 | 0 |
| C01 | 4.23 | 62.6 | 0 |
| C02 | 62.96 | 3.00 | 2.58 |
| C03 | 64.31 | 2.03 | 2.27 |
| C04 | 66.39 | 2.65 | 2.44 |
| C05 | 59.73 | 3.35 | 3.6 |
| C06 | 60.27 | 1.36 | 2.45 |

## 5. Supplementary Table S2. Pre21 RNA2 Conjugation Data

**Table S2.** Small molecule disulfide screening data for pre21 RNA2.

| | % Relative Abundances | | |
|---|---|---|---|
| **Compound** | **Thiol** | **N,N-DMA** | **Target** |
| A01 | 6.72 | 68.03 | 1.94 |
| A02 | 2.51 | 76.25 | 2.25 |
| A03 | 8.57 | 69.06 | 0 |
| A04 | 0 | 44.96 | 32.05 |
| A05 | 0 | 74.56 | 13.63 |
| A06 | 3.23 | 49.22 | 5.04 |
| A07 | 15.24 | 74.14 | 0 |
| A08 | 0 | 73.5 | 0 |
| A09 | 35.1 | 5.32 | 0 |
| A10 | 81.9 | 2.25 | 0 |
| A11 | 72.58 | 5.04 | 2.3 |
| A12 | 20.46 | 44.1 | 0 |
| B01 | 7.65 | 63.77 | 0:00 |
| B02 | 55.58 | 23.04 | 1.87 |
| B03 | 29.9 | 28.09 | 0 |
| B04 | 72.9 | 8.17 | 0 |
| B05 | 76.5 | 4.02 | 0 |
| B06 | 77.1 | 1.68 | 0 |
| B07 | 76.8 | 1.48 | 0 |
| B08 | 78 | 6.34 | 0 |
| B09 | 80.2 | 2.02 | 0 |
| B10 | 69.3 | 1.92 | 0 |
| B11 | 71.87 | 2.7 | 1.75 |
| B12 | 51.9 | 2.96 | 0 |
| C01 | 30.66 | 39.92 | 3.24 |
| C02 | 54.16 | 2.99 | 1.25 |
| C03 | 40.11 | 3.11 | 0 |
| C04 | 39.21 | 3.5 | 1.65 |
| C05 | 31.88 | 6.33 | 1.59 |
| C06 | 55.1 | 2.63 | 1.67 |

## 6. Pre21 RNA1 Conjugation Data Mass Spectra

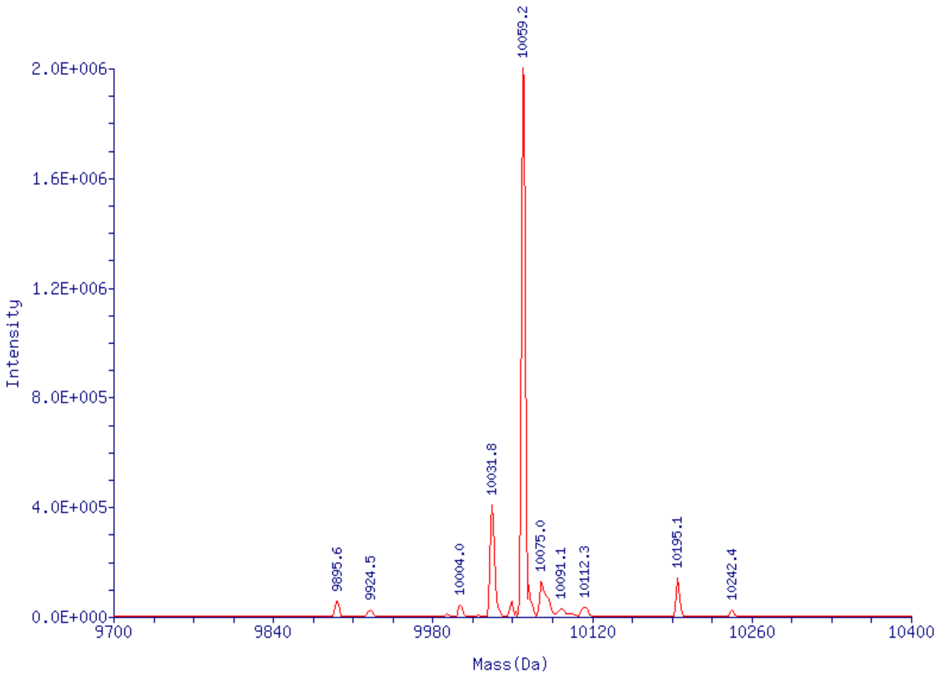


**Compound A01.**

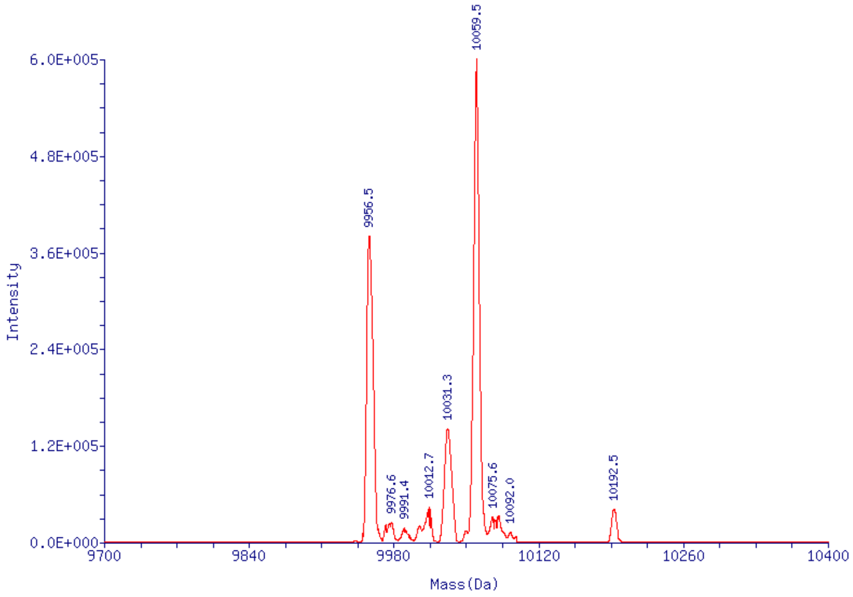


**Compound A02.**

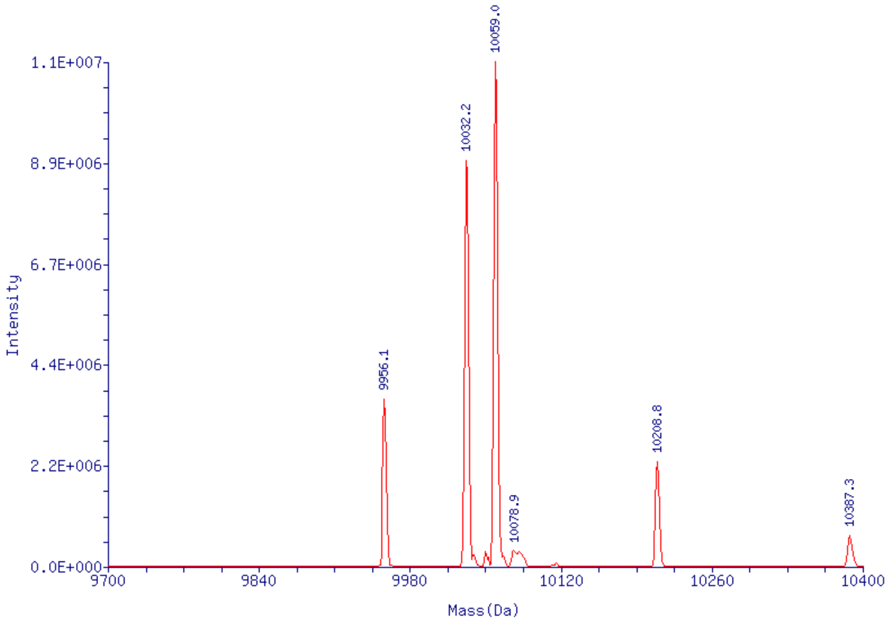


**Compound A03**.

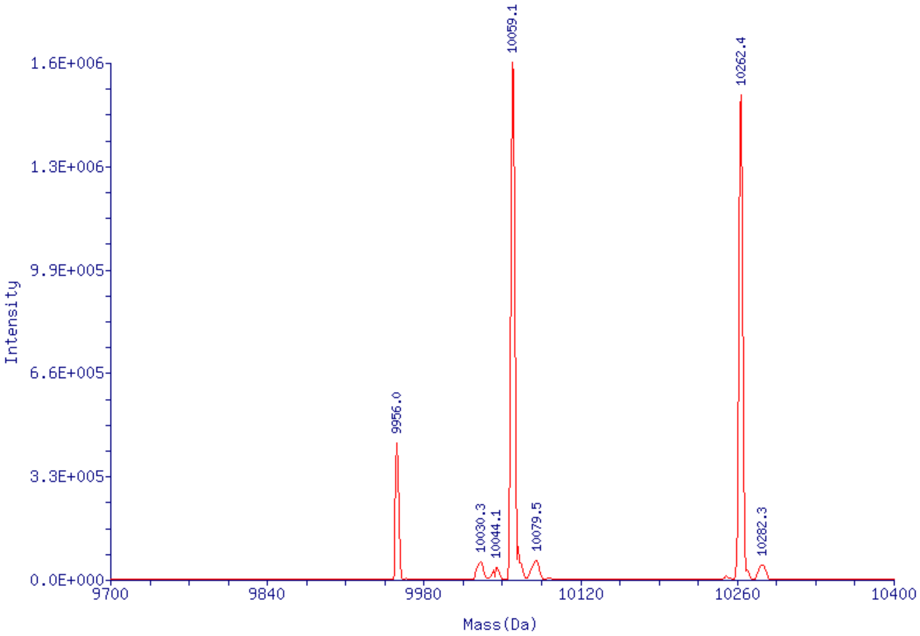


**Compound A04**.

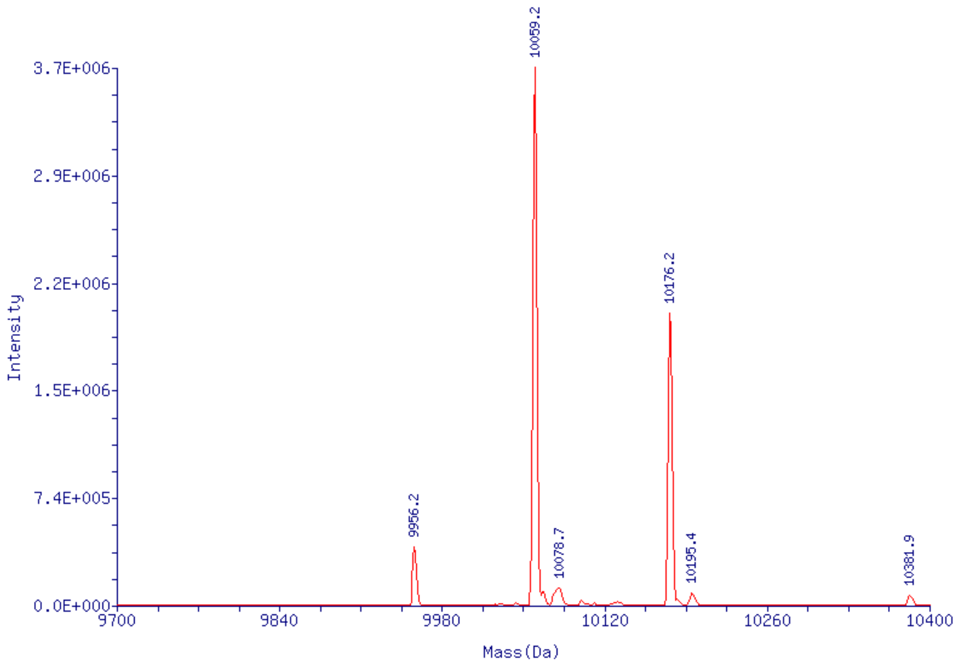


**Compound A05**.

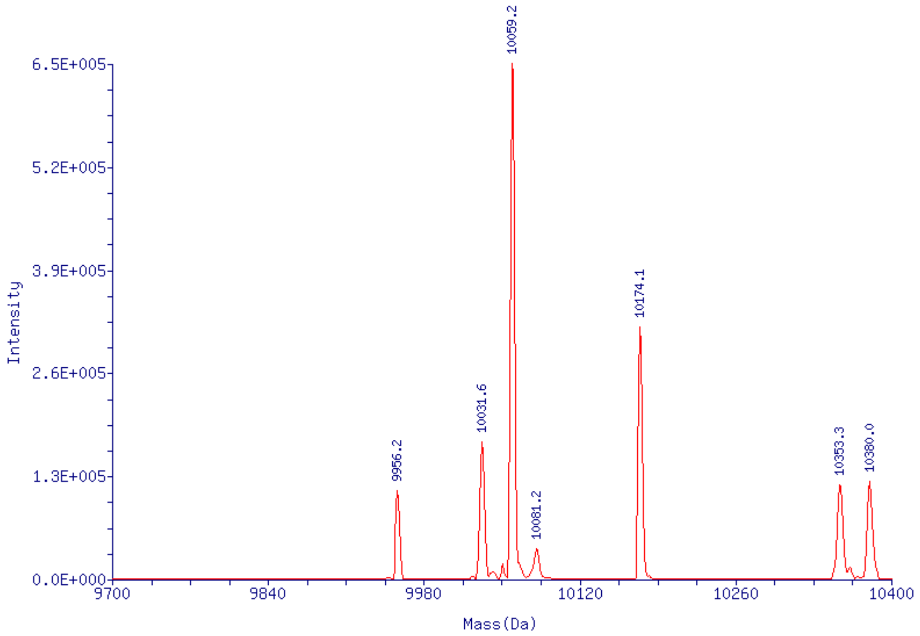


**Compound A06**.

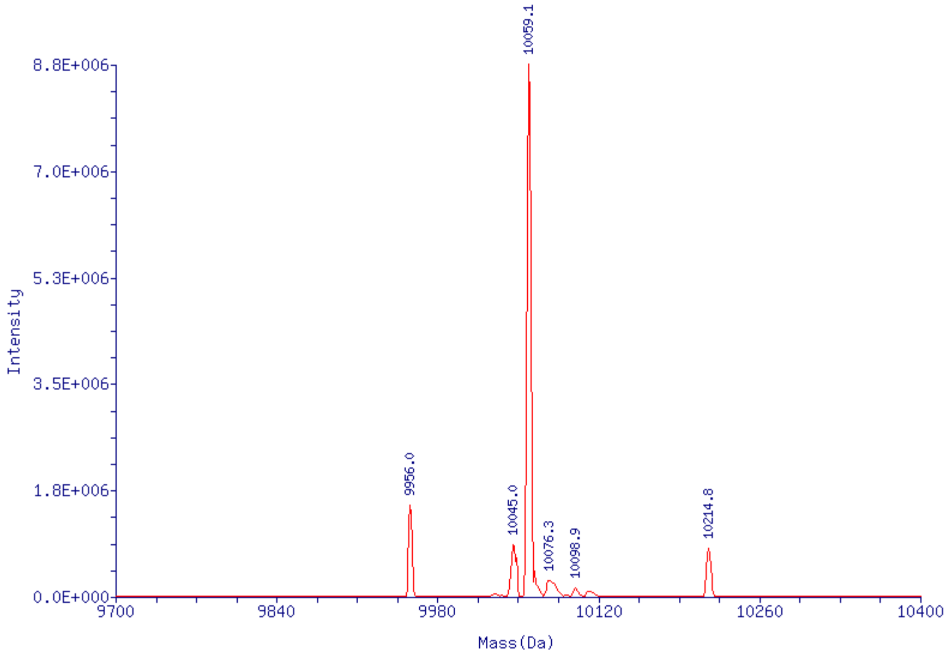


**Compound A07**.

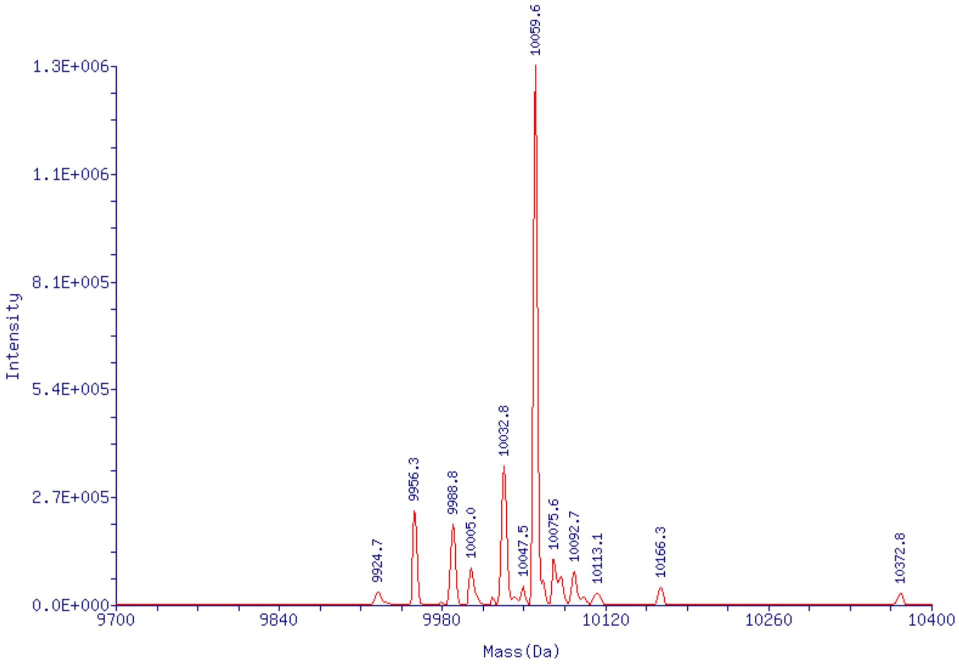


**Compound A08**.

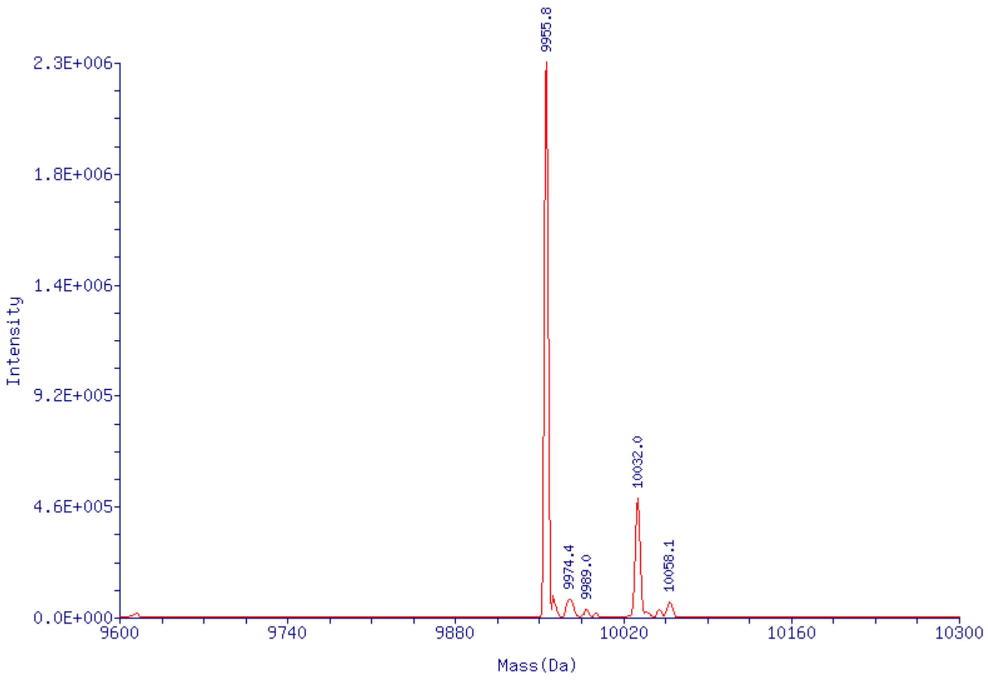


**Compound A09**.

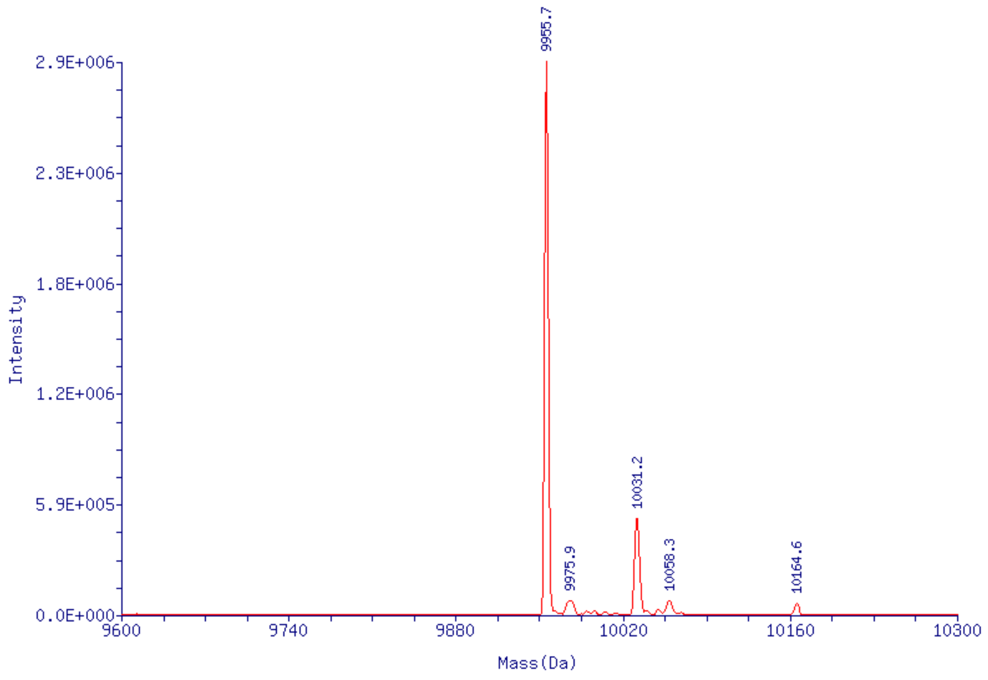


**Compound A10**.

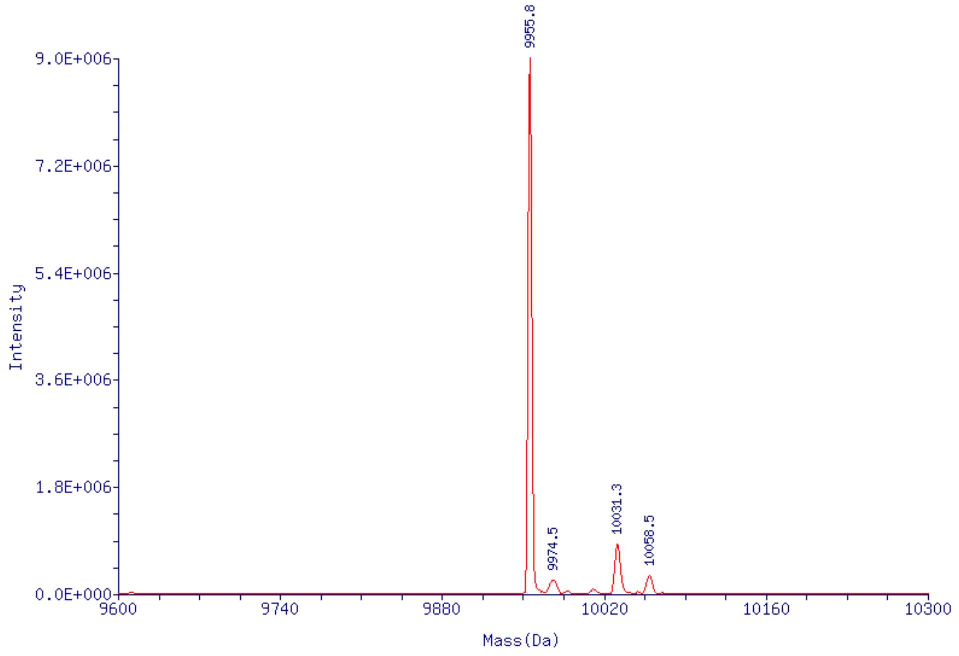


**Compound A11.**

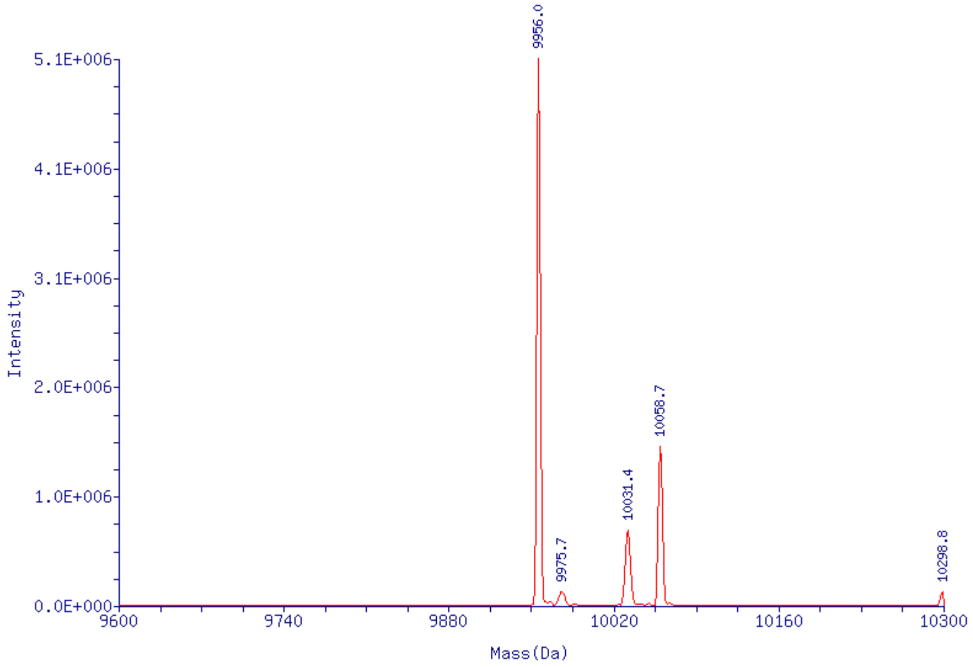


**Compound A12**.

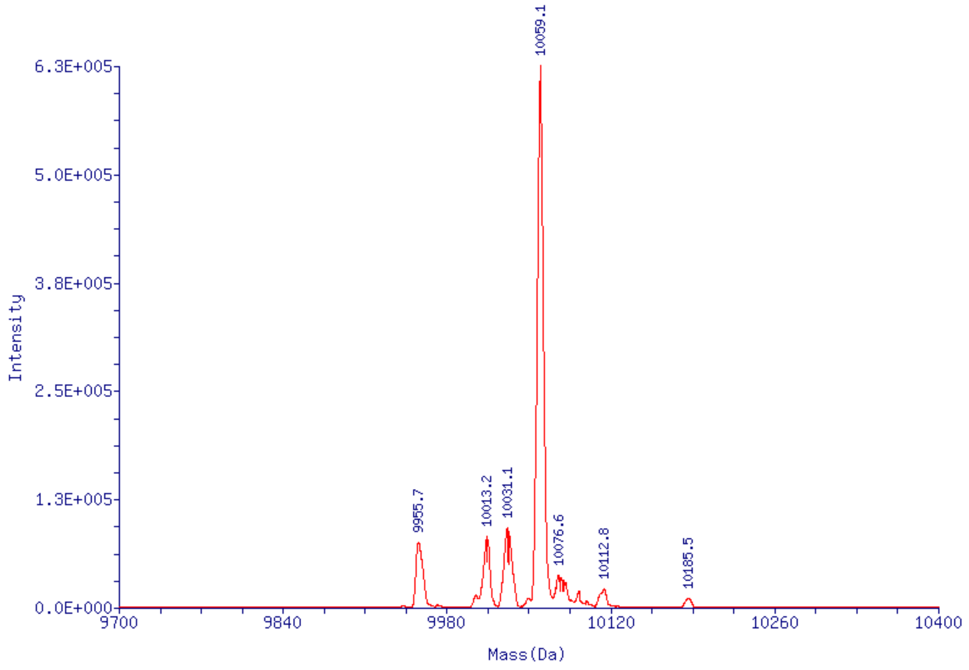


**Compound B01**.

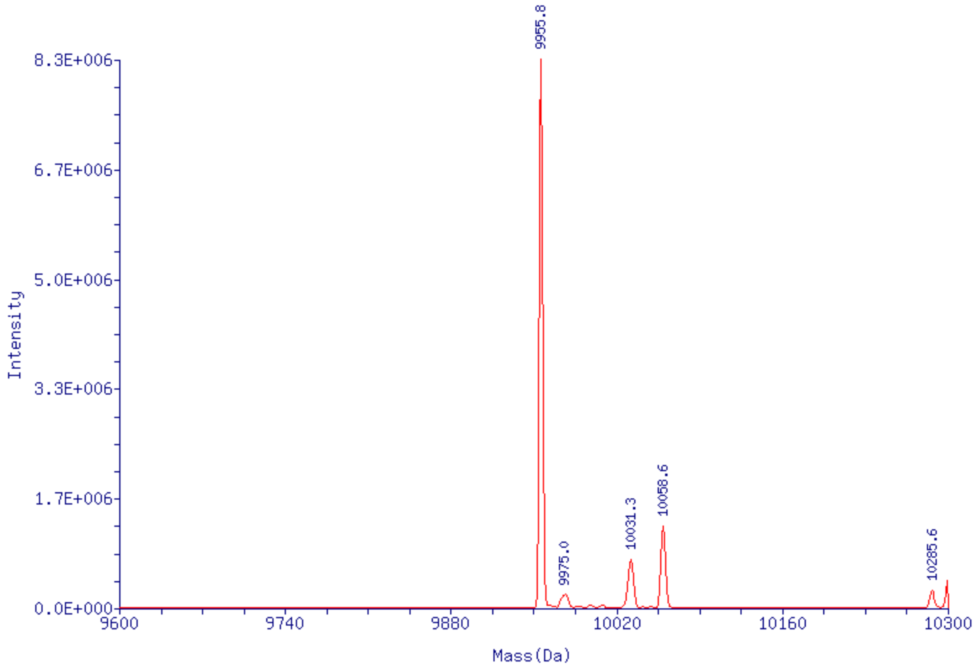


**Compound B02**.

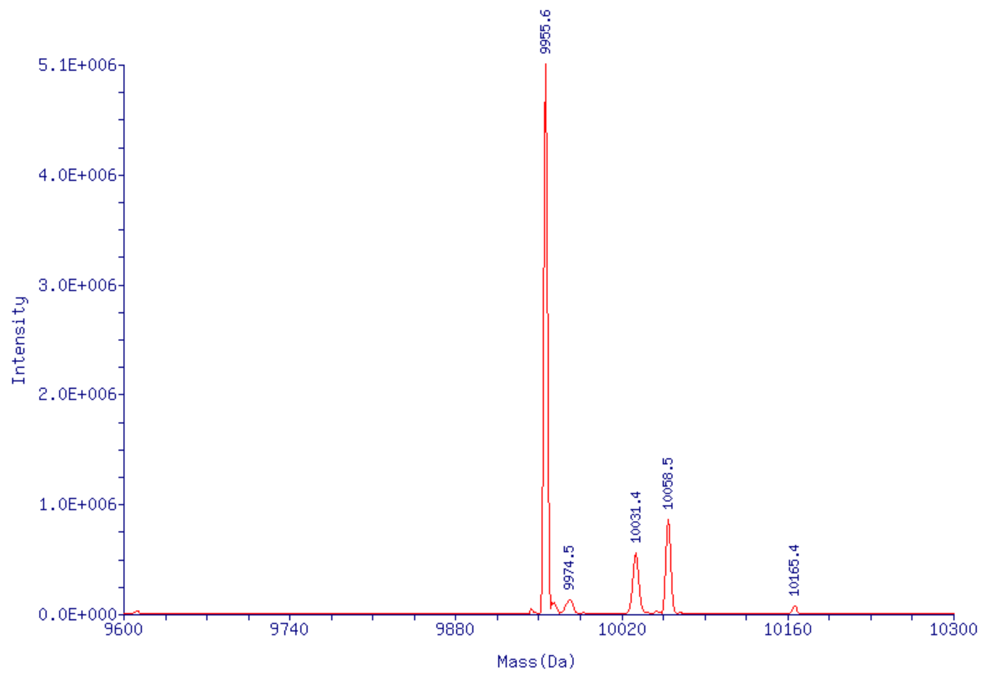


**Compound B03**.

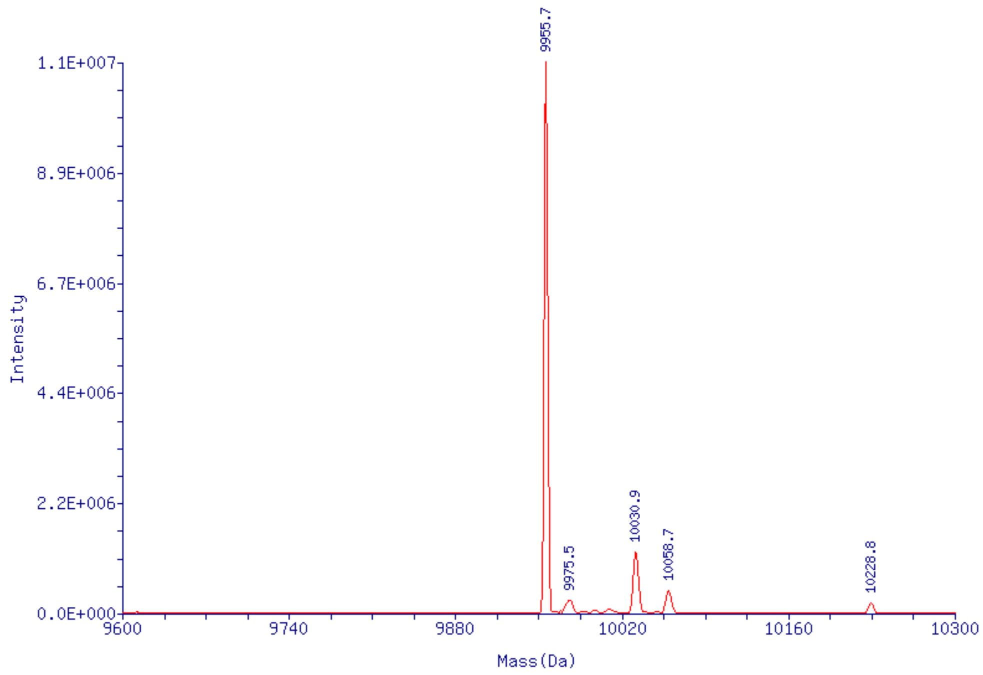


**Compound B04**.

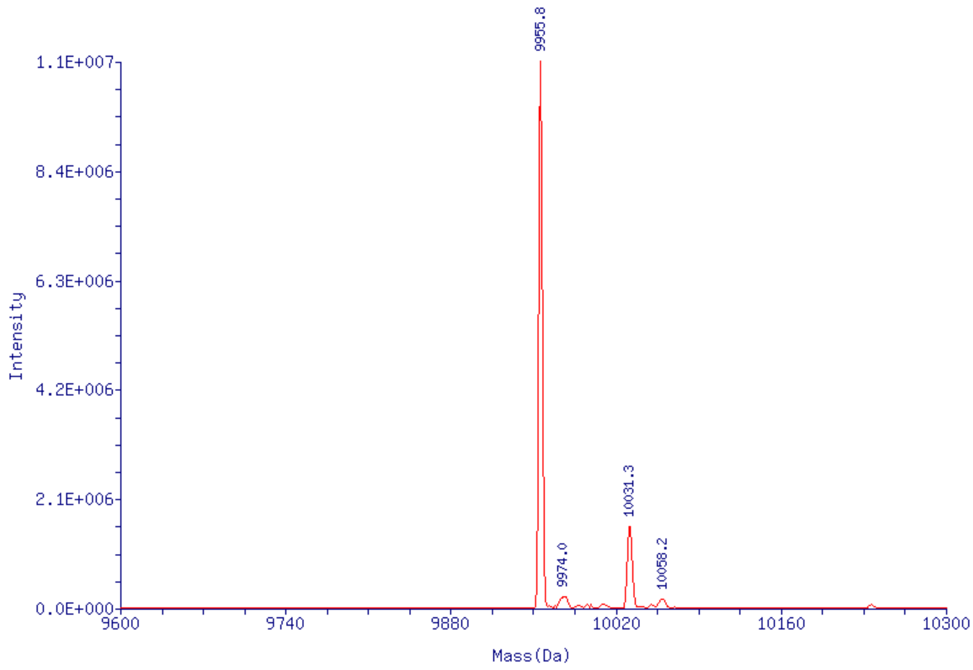


**Compound B05.**

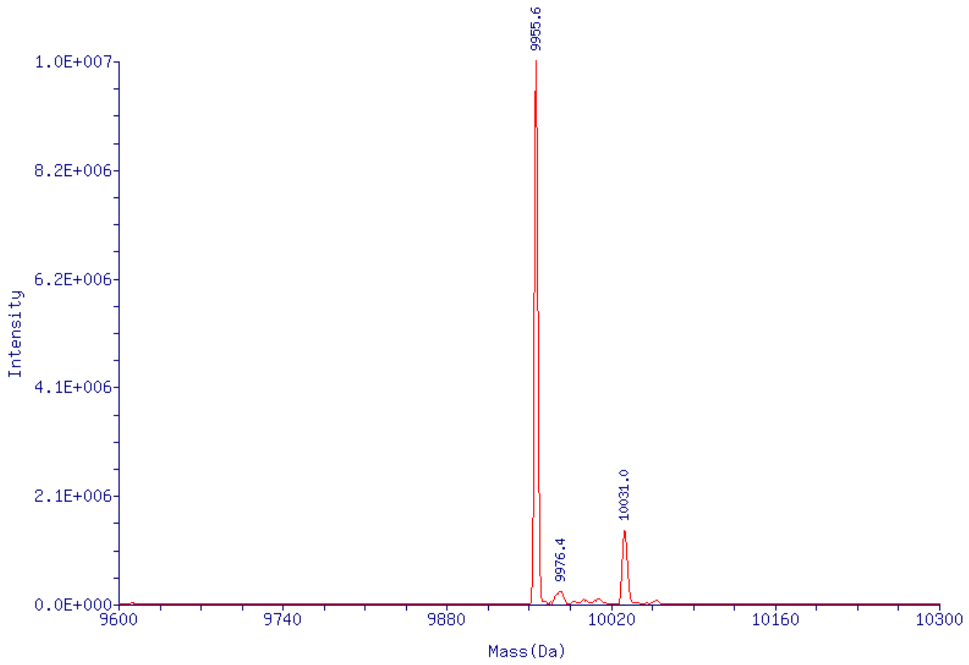


**Compound B06.**

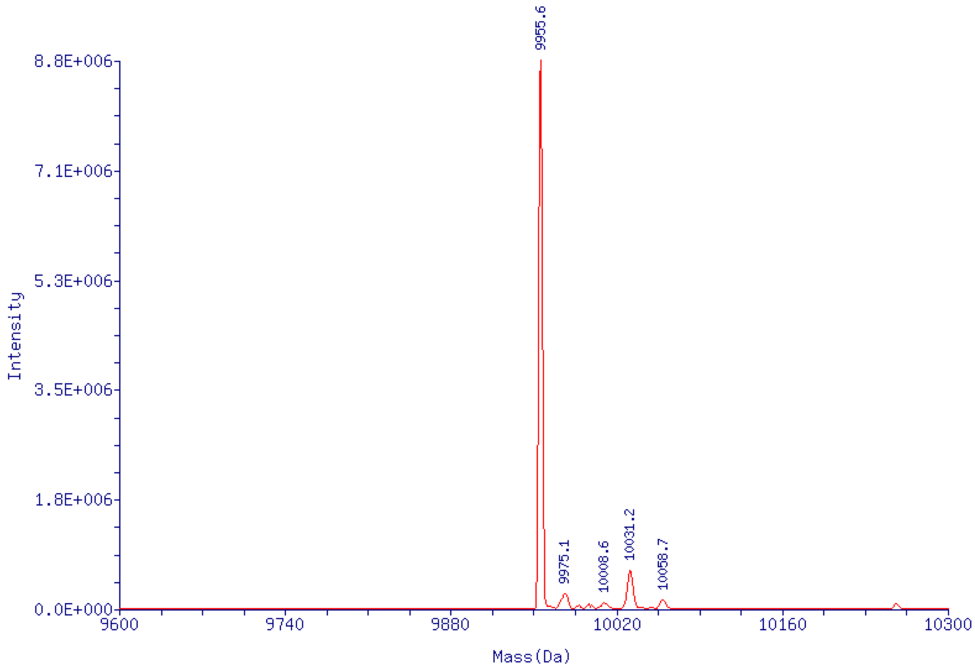


**Compound B07**.

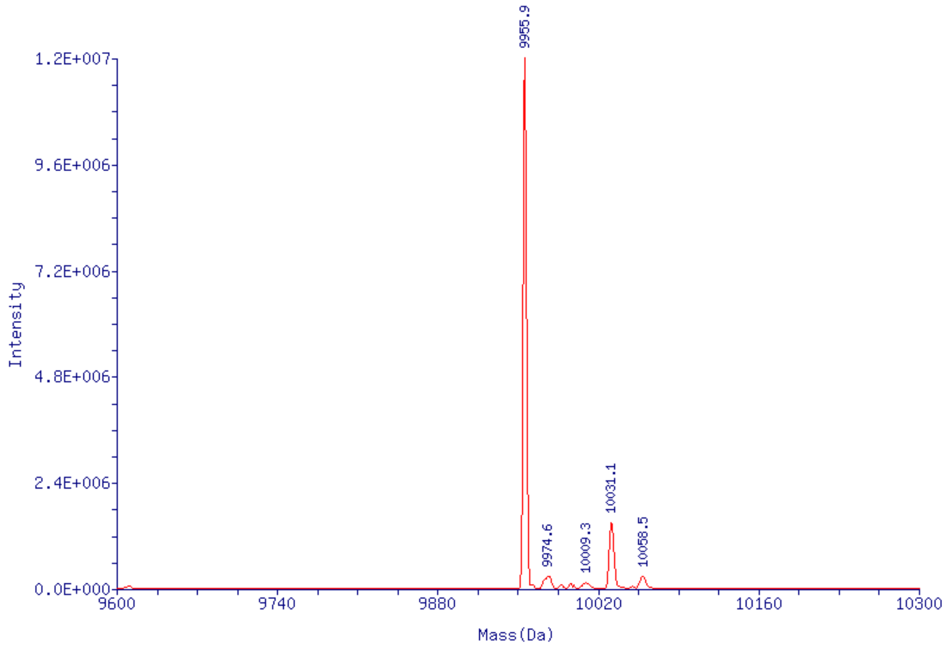


**Compound B08**.

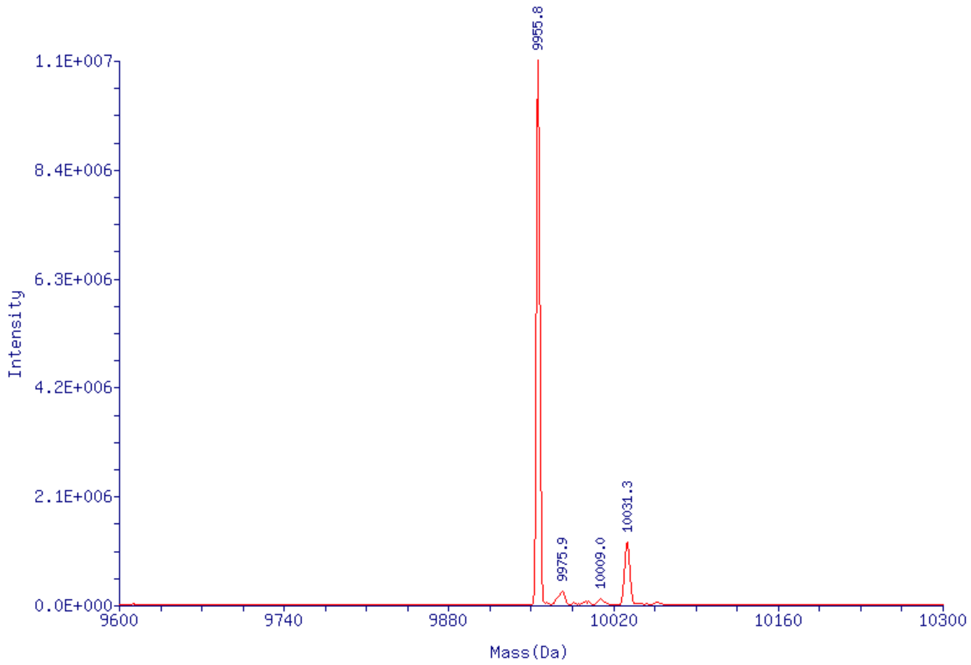


**Compound B09**.

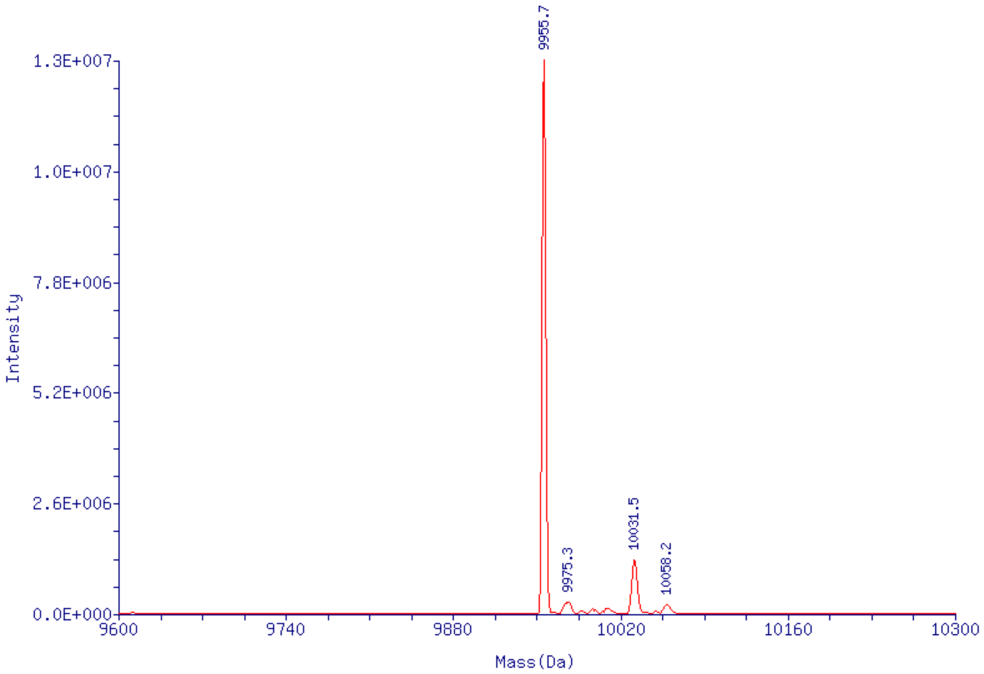


**Compound B10**.

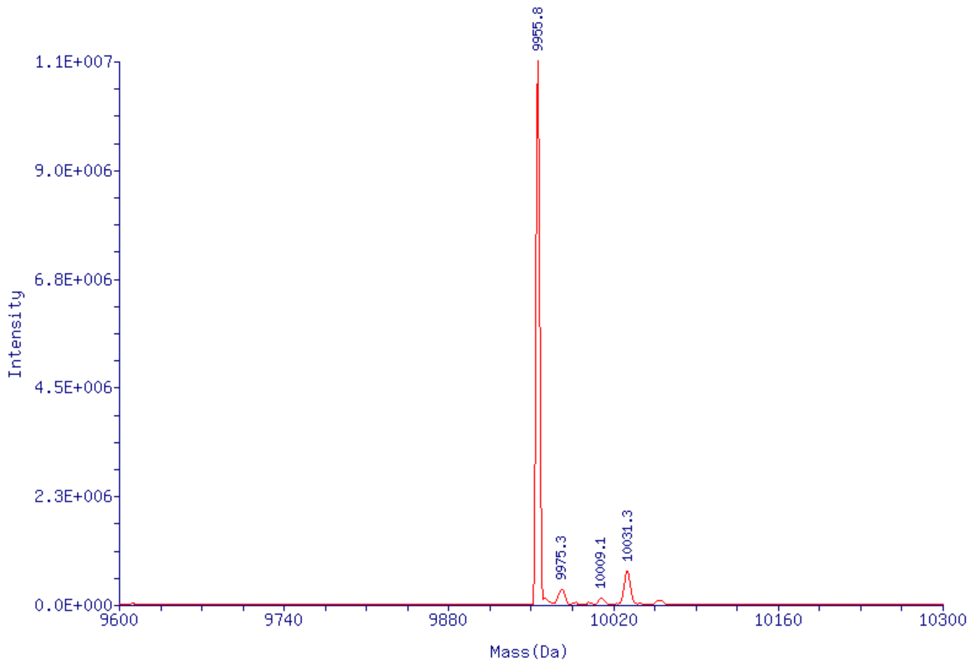


**Compound B11**.

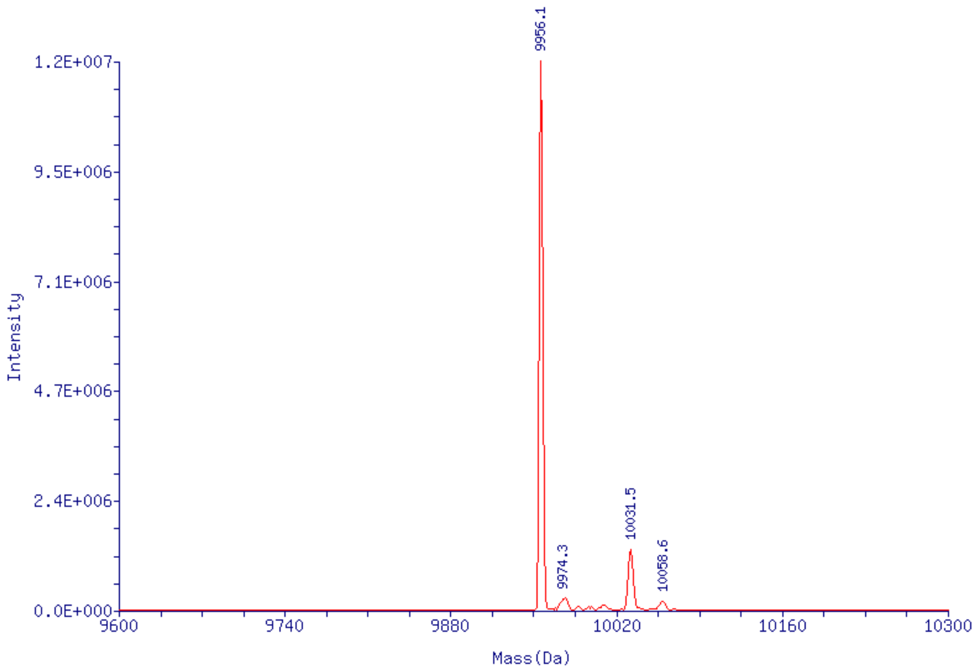


**Compound B12**.

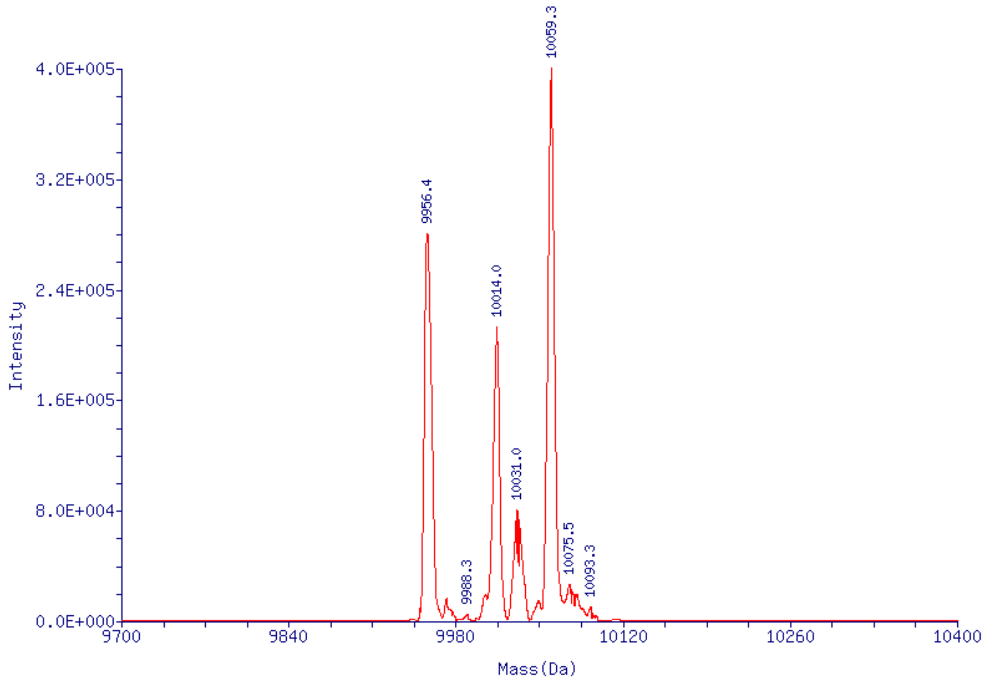


**Compound C01**.

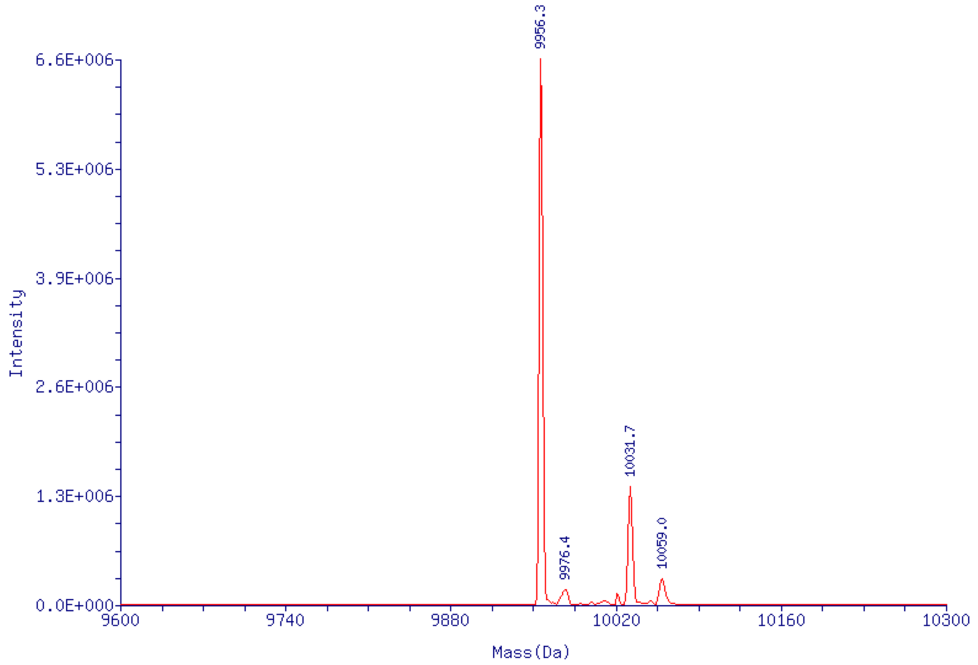


**Compound C02**.

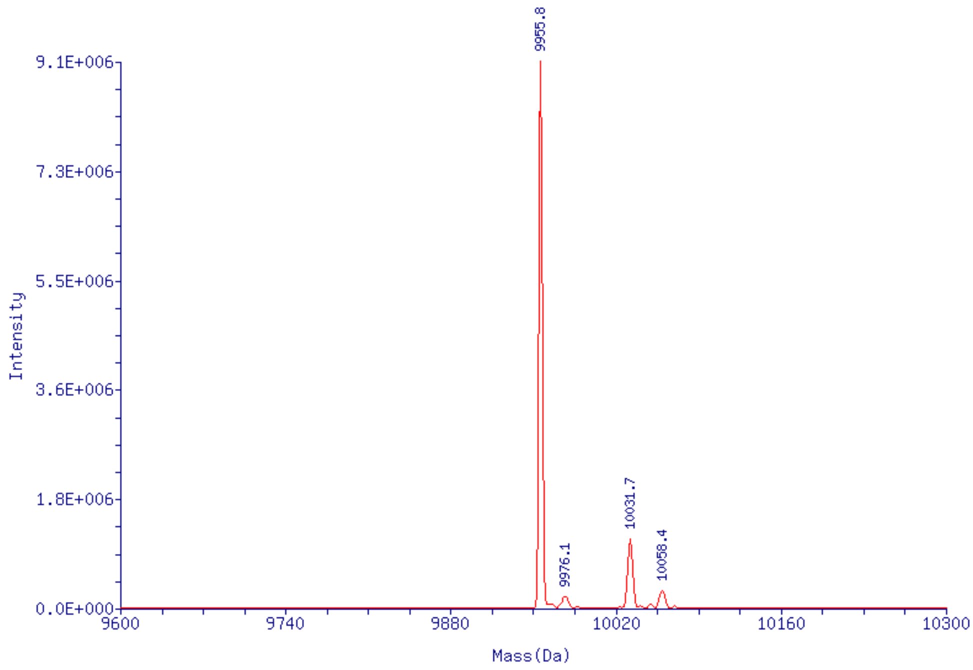


**Compound C03**.

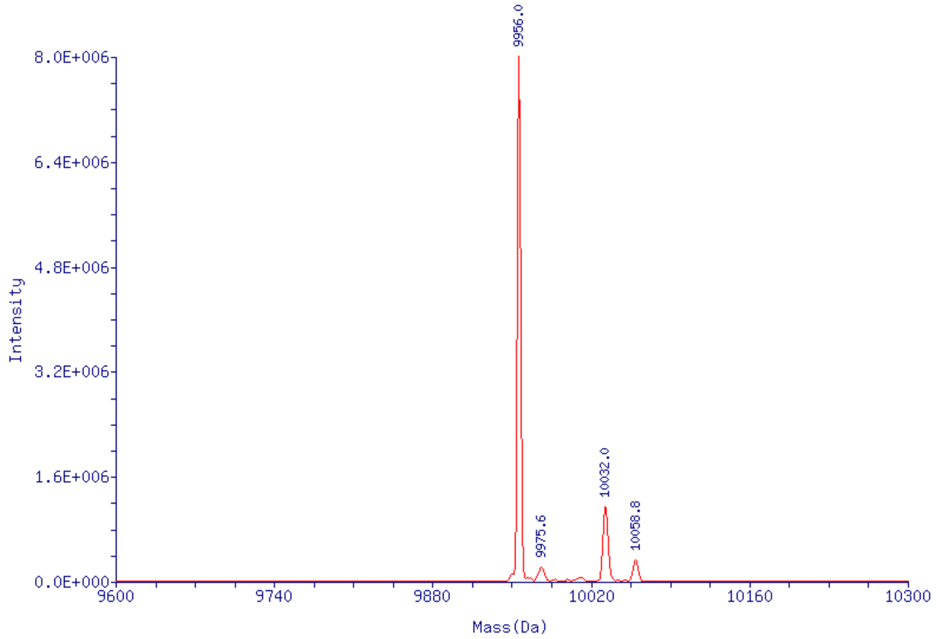


**Compound C04**.

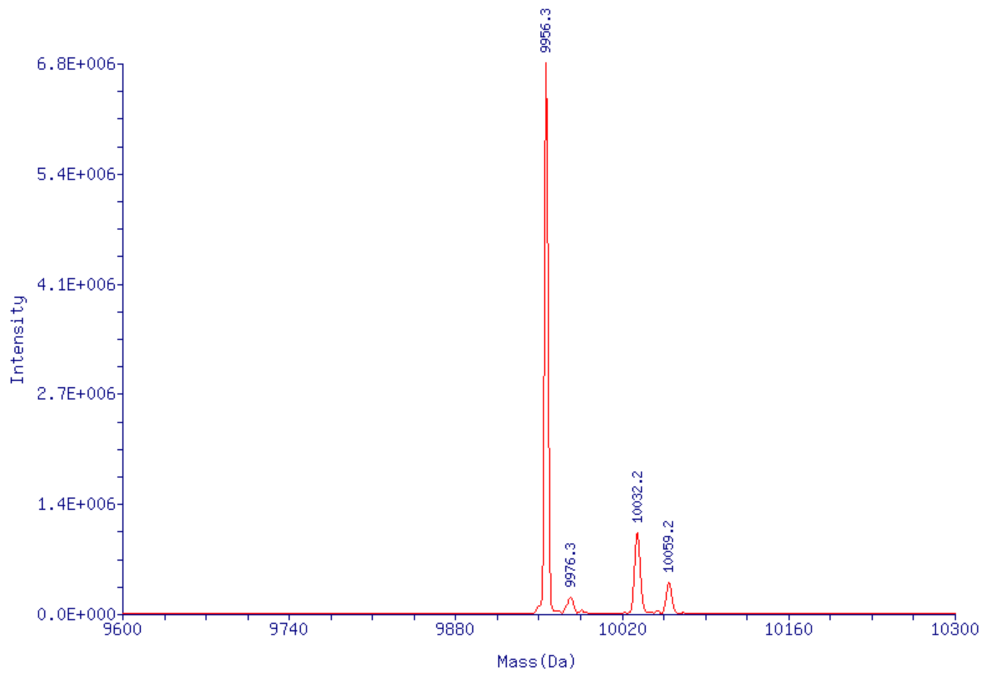


**Compound C05**.

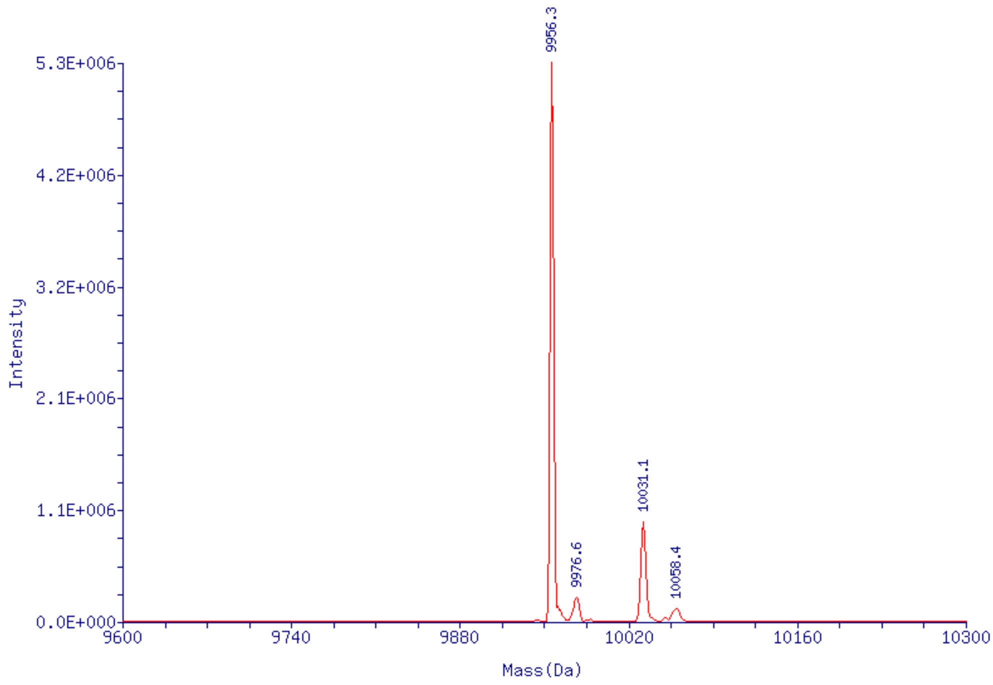


**Compound C06**.

## 7. Pre21 RNA2 Conjugation Data Mass Spectra.

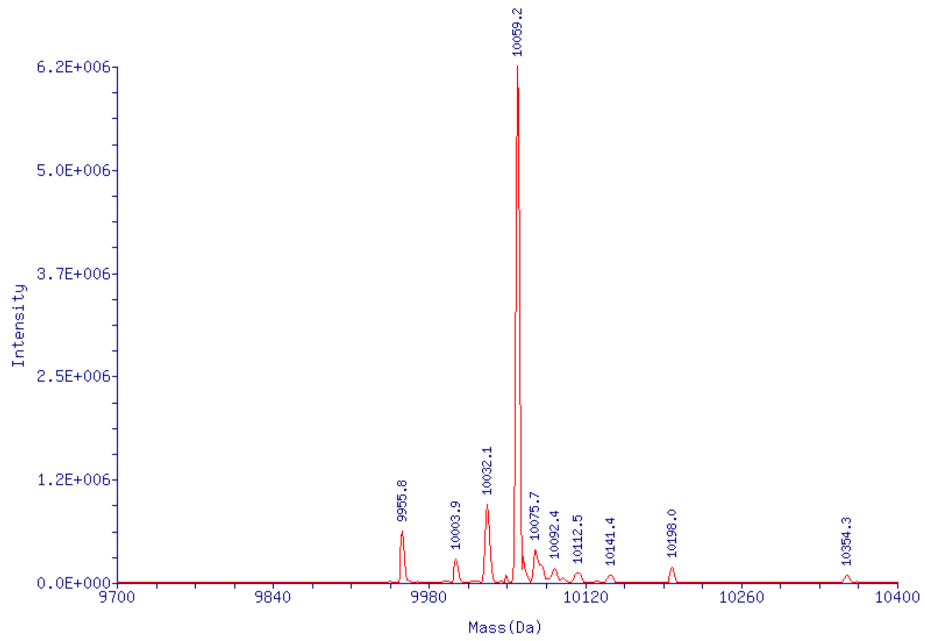


**Compound A01**.

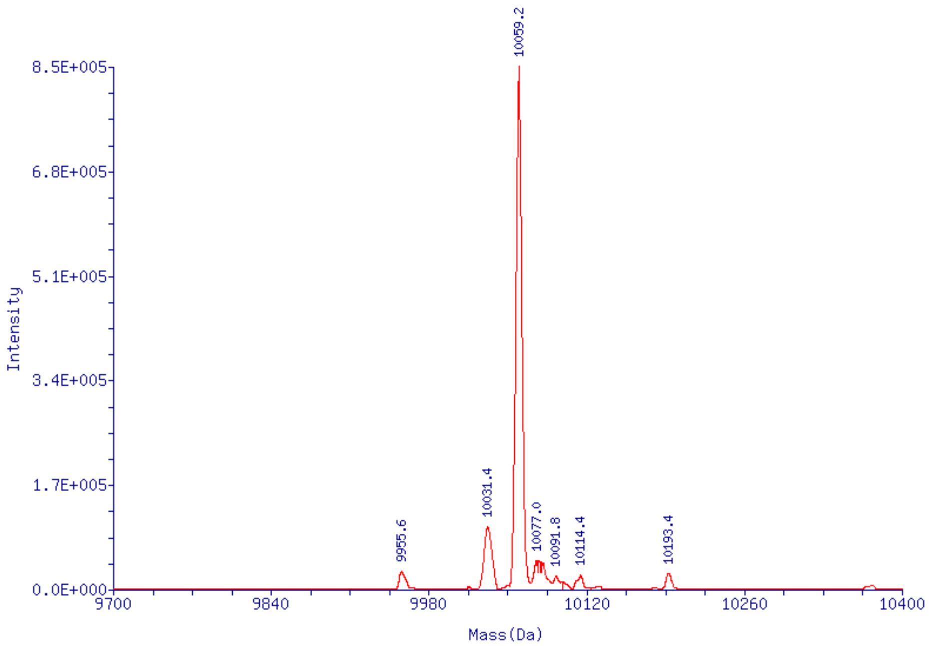


**Compound A02**.

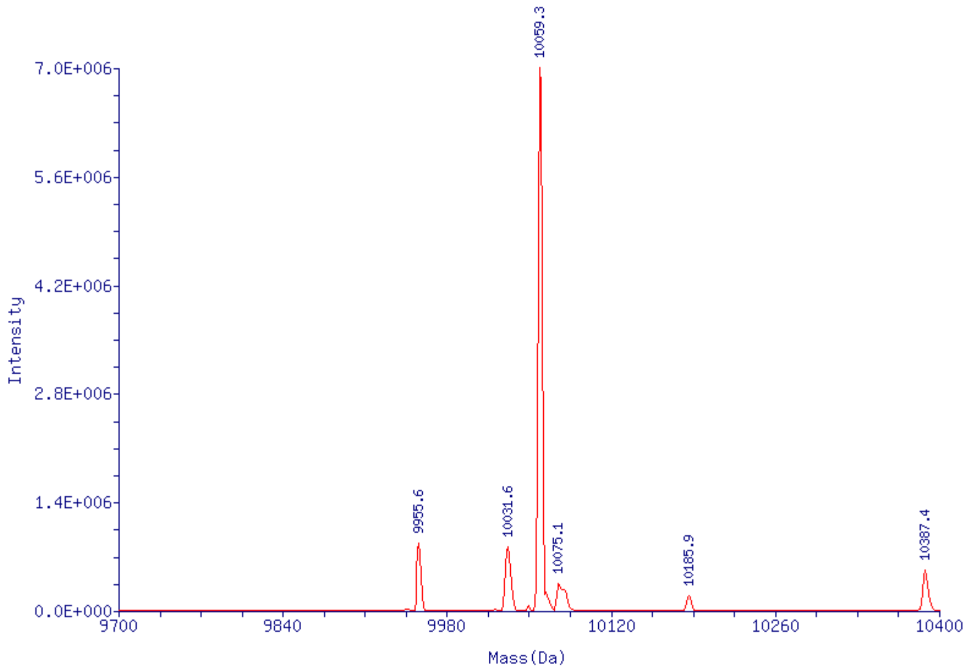


**Compound A03**.

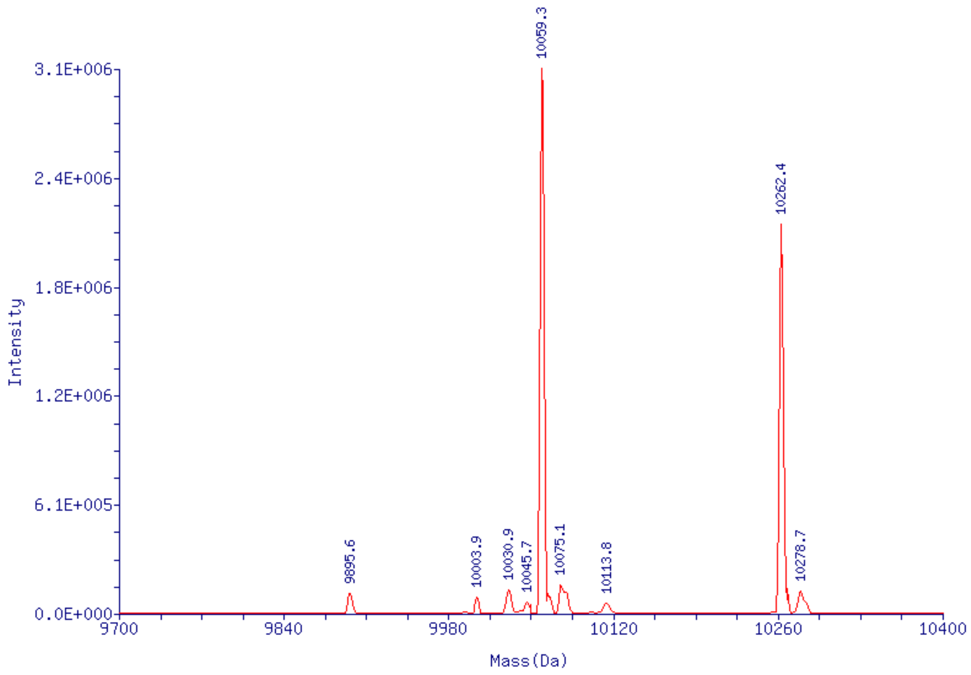


**Compound A04**.

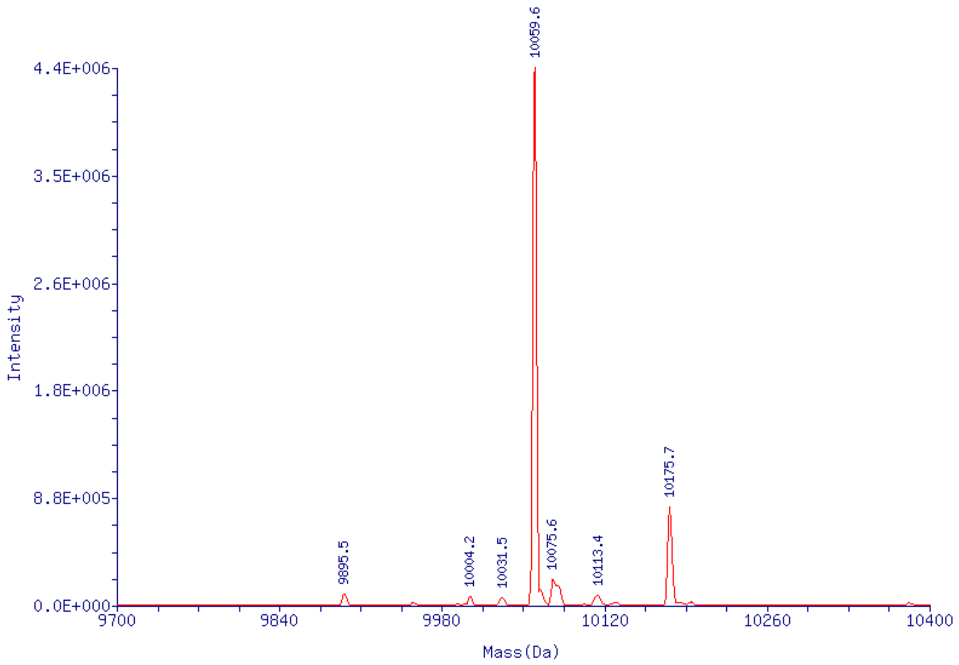


**Compound A05**.

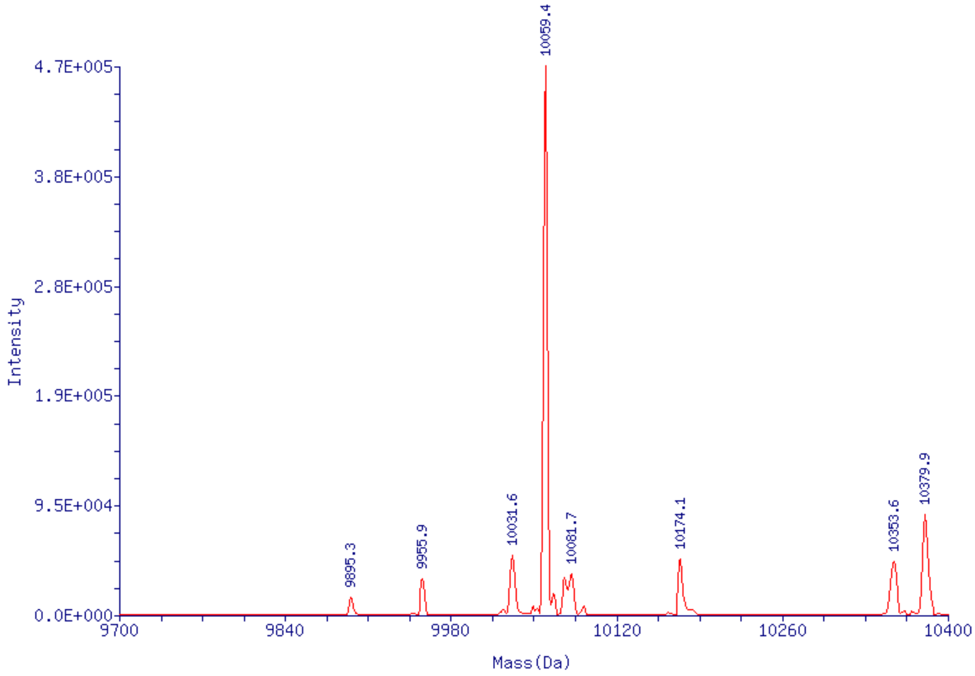


**Compound A06**.

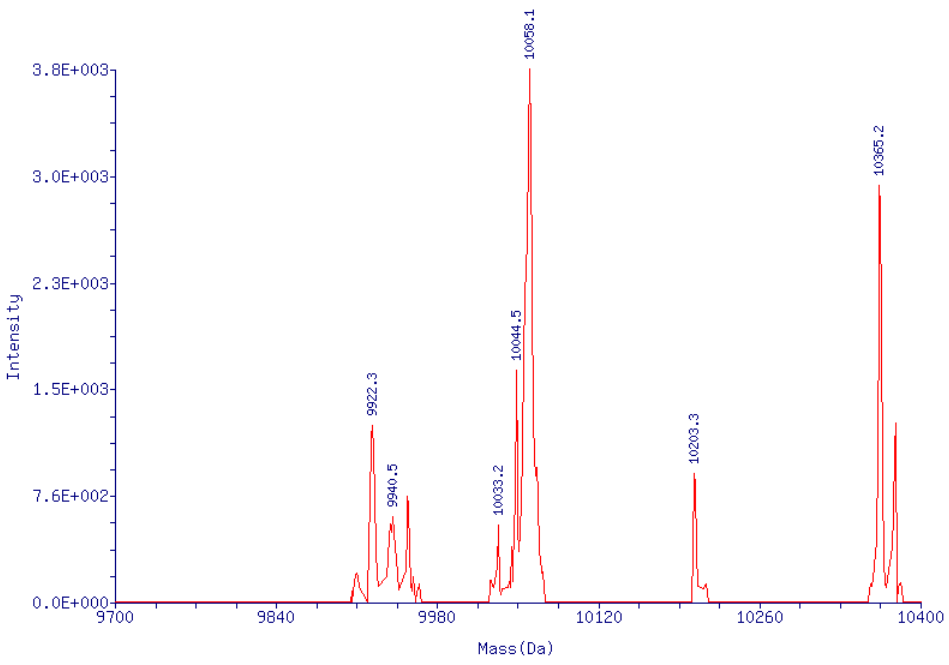


**Compound A07**.

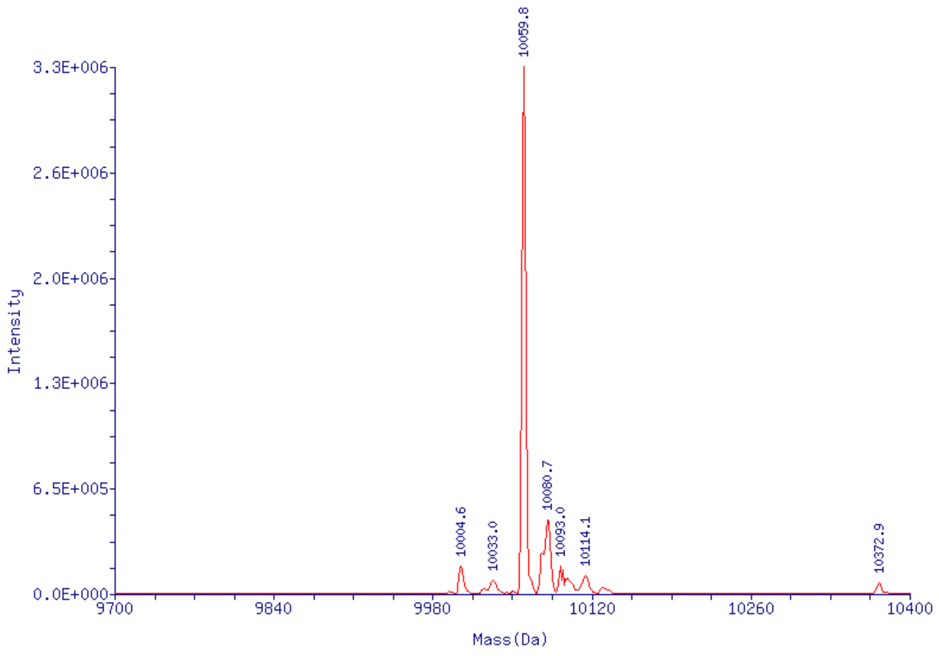


**Compound A08**.

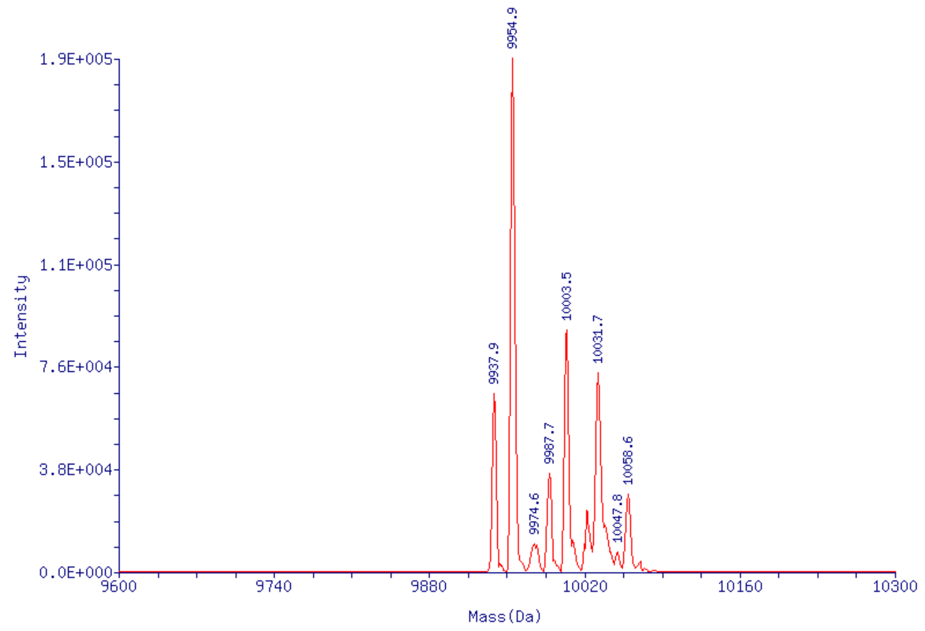


**Compound A09**.

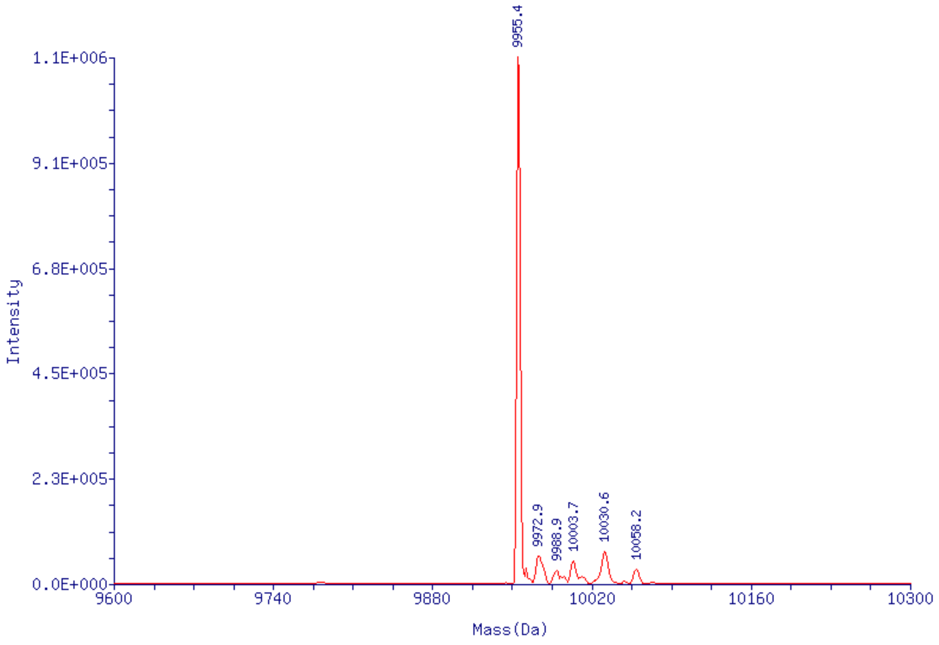


**Compound A10**.

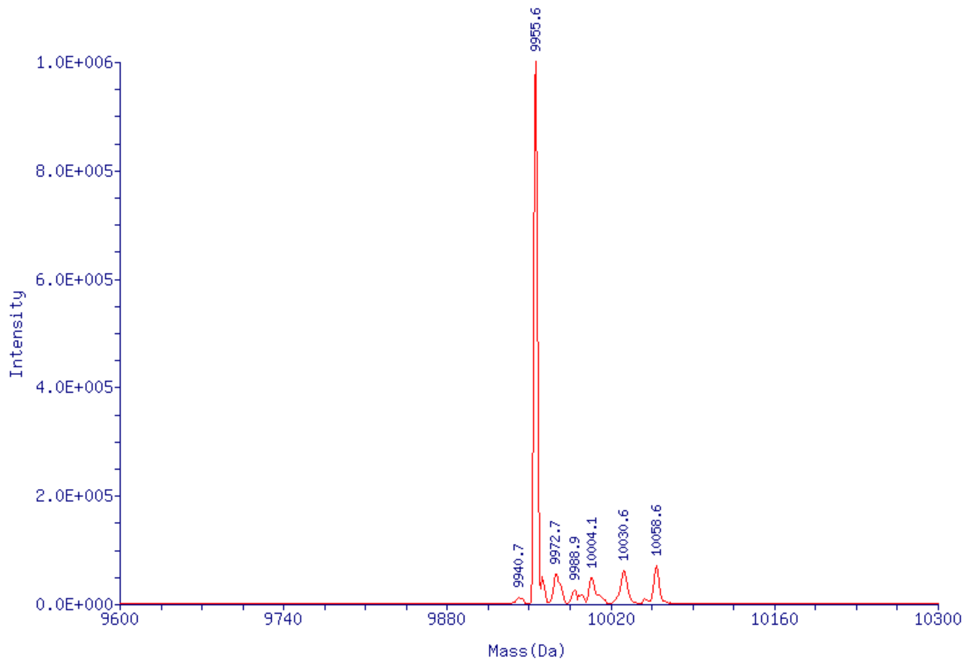


**Compound A11**.

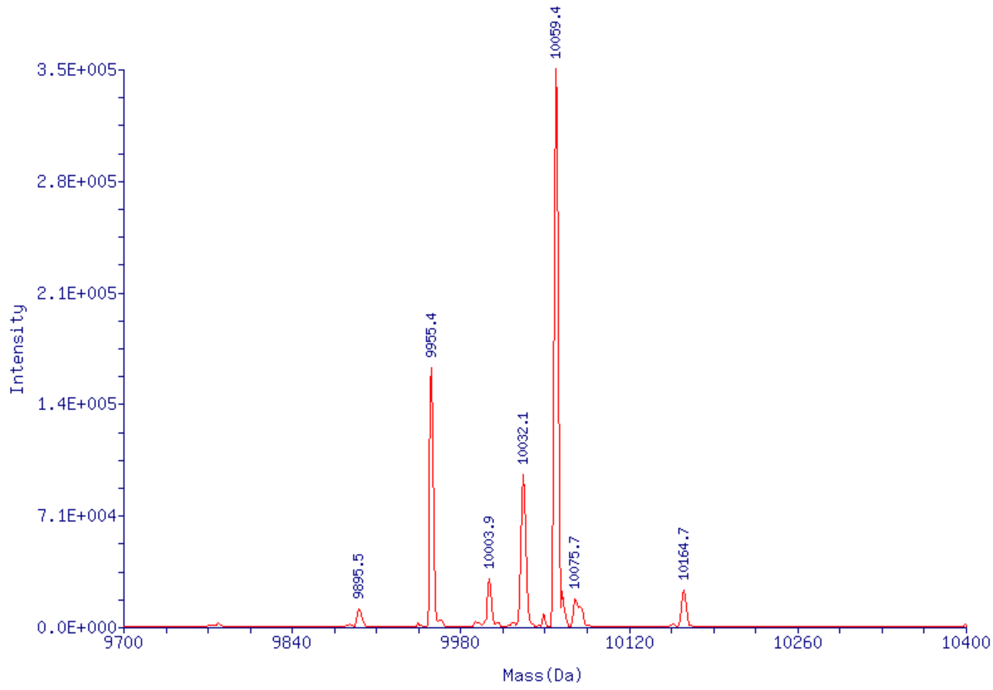


**Compound A12**.

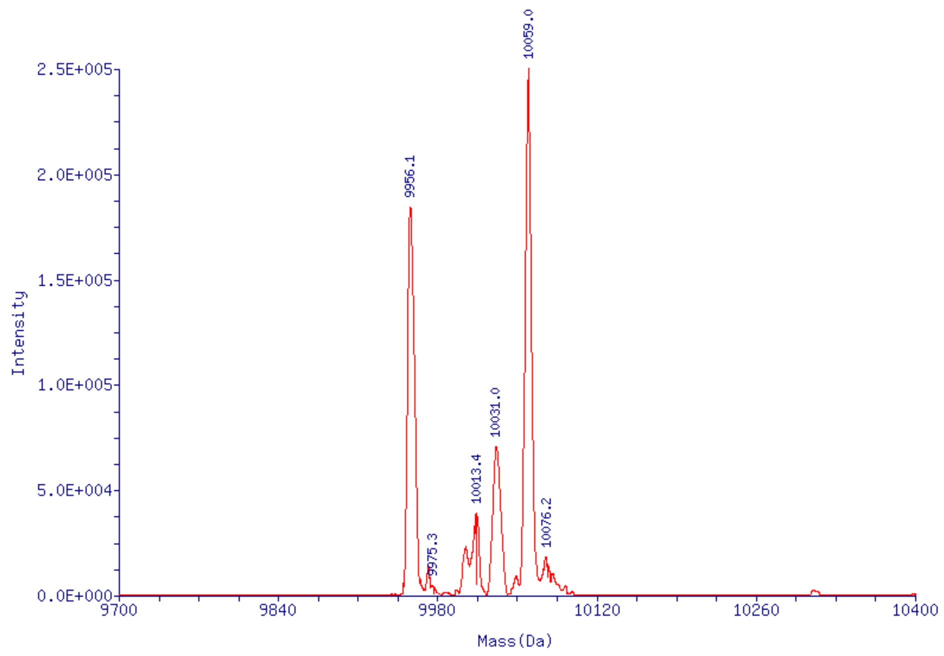


**Compound B01**.

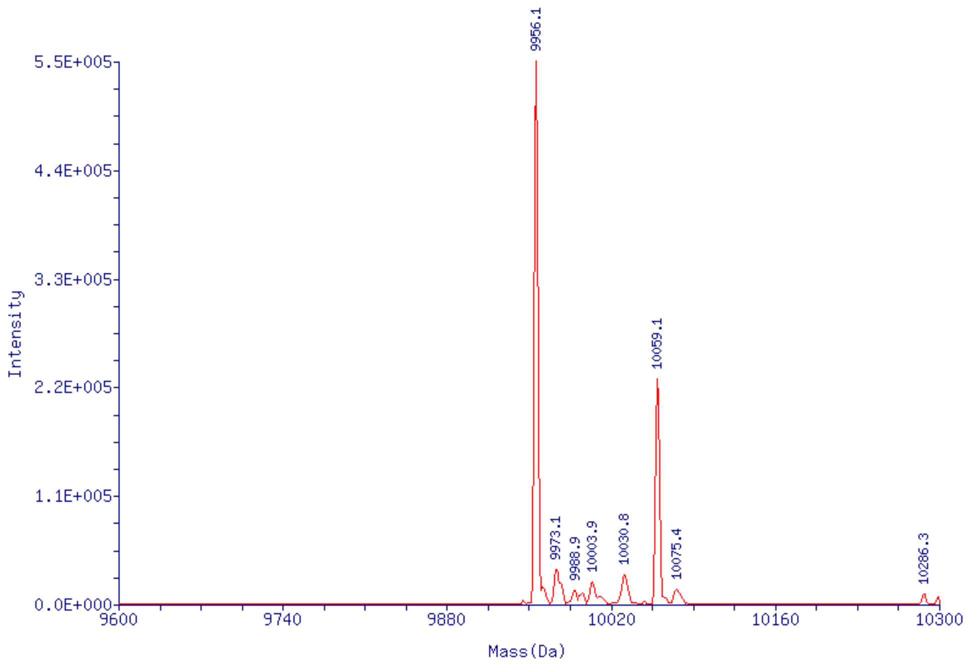


**Compound B02**.

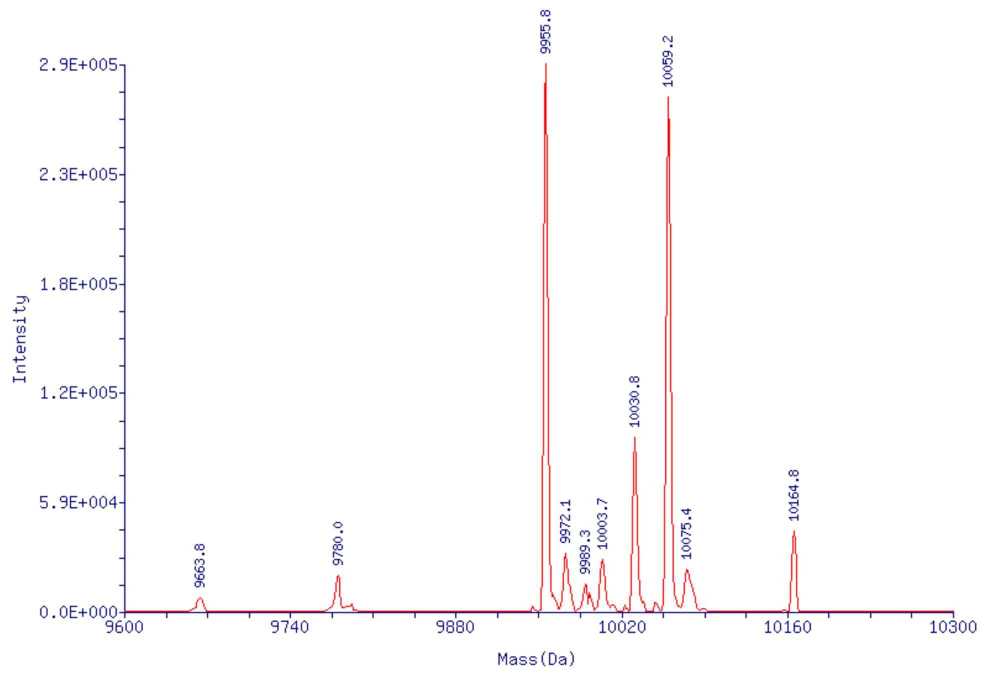


**Compound B03**.

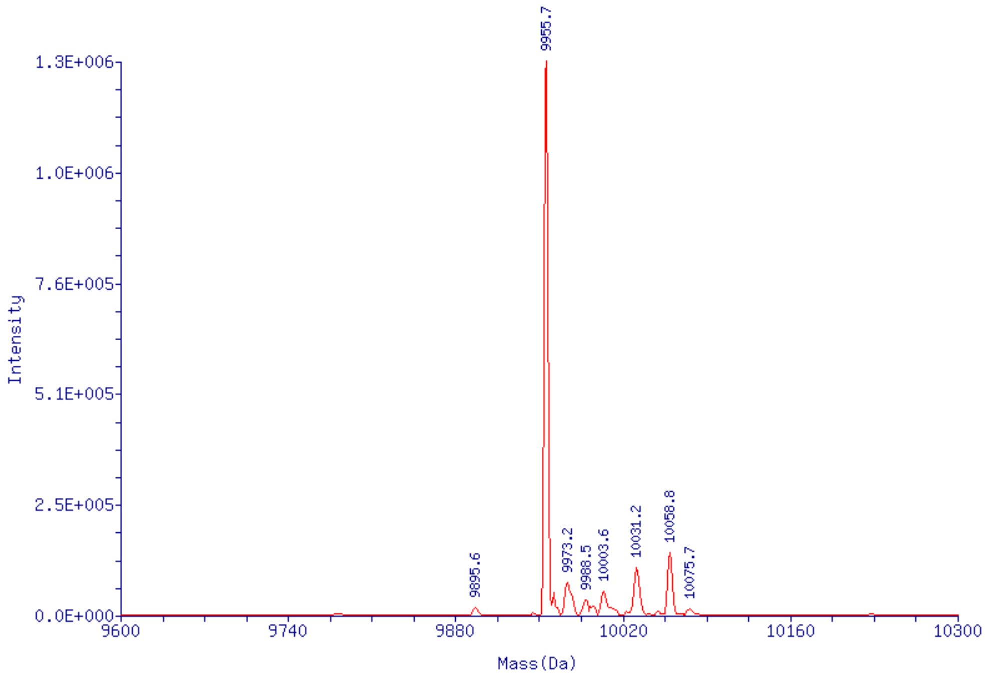


**Compound B04**.

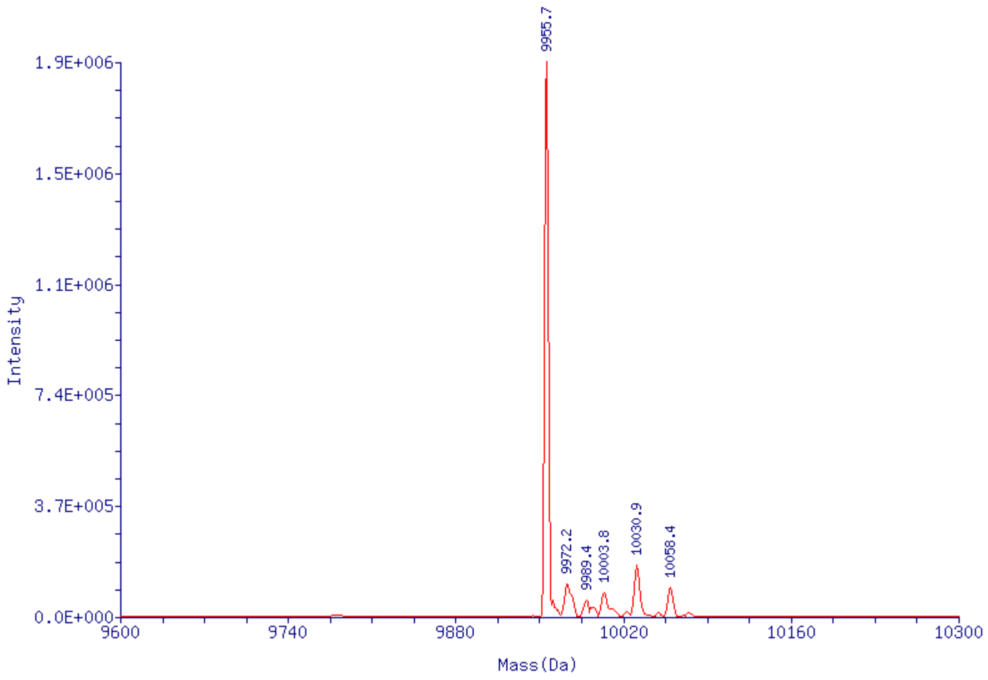


**Compound B05**.

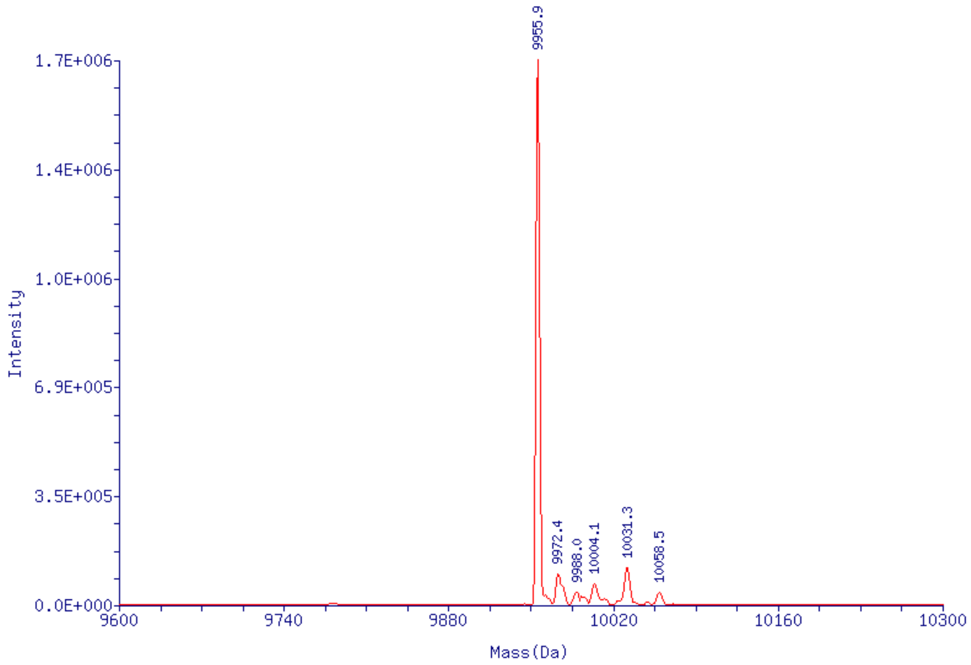


**Compound B06**.

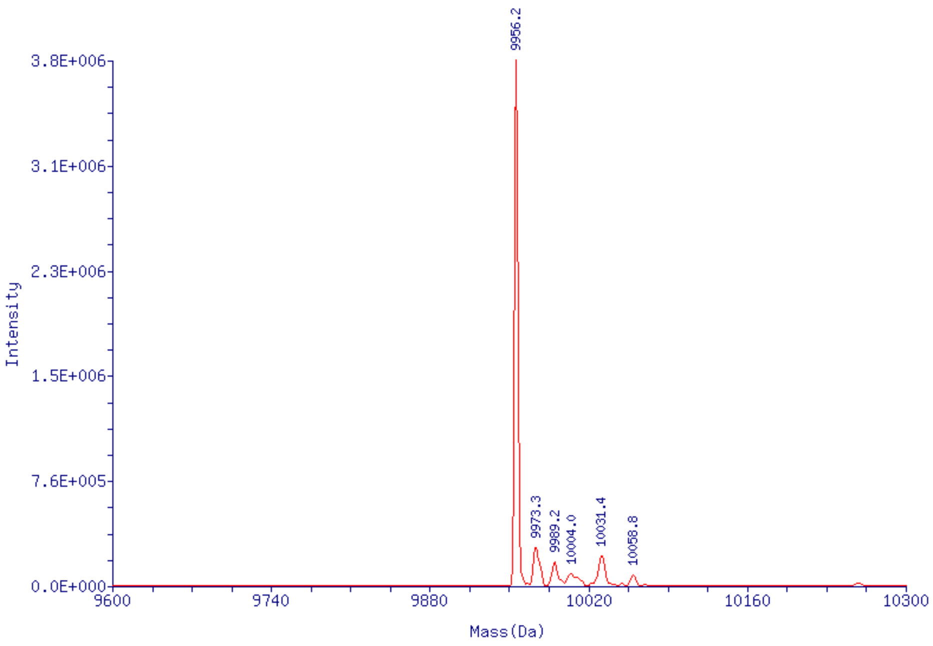


**Compound B07**.

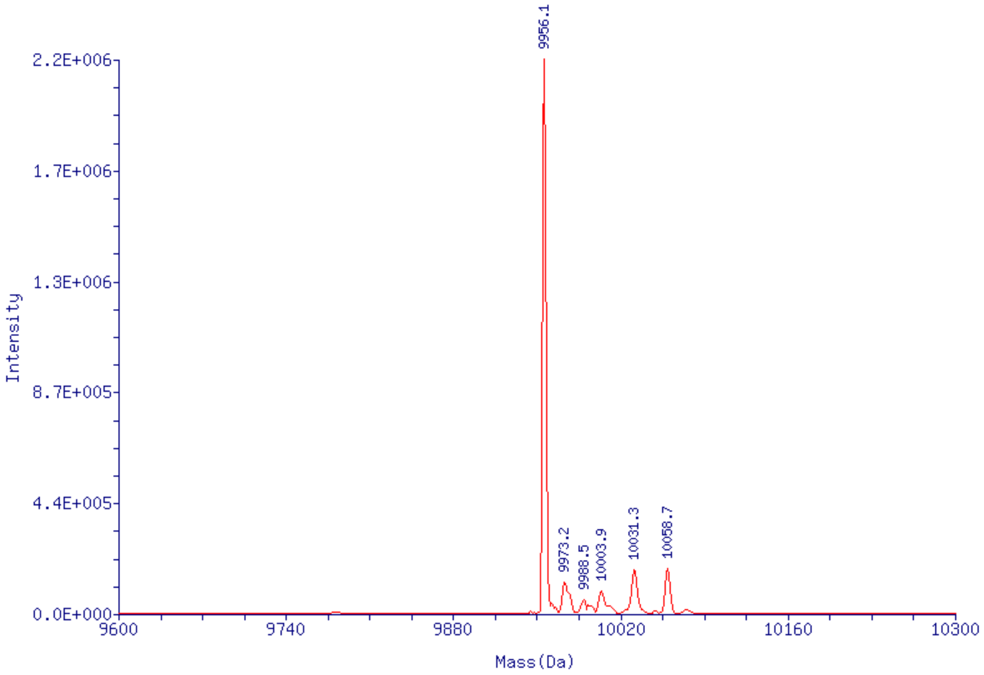


**Compound B08**.

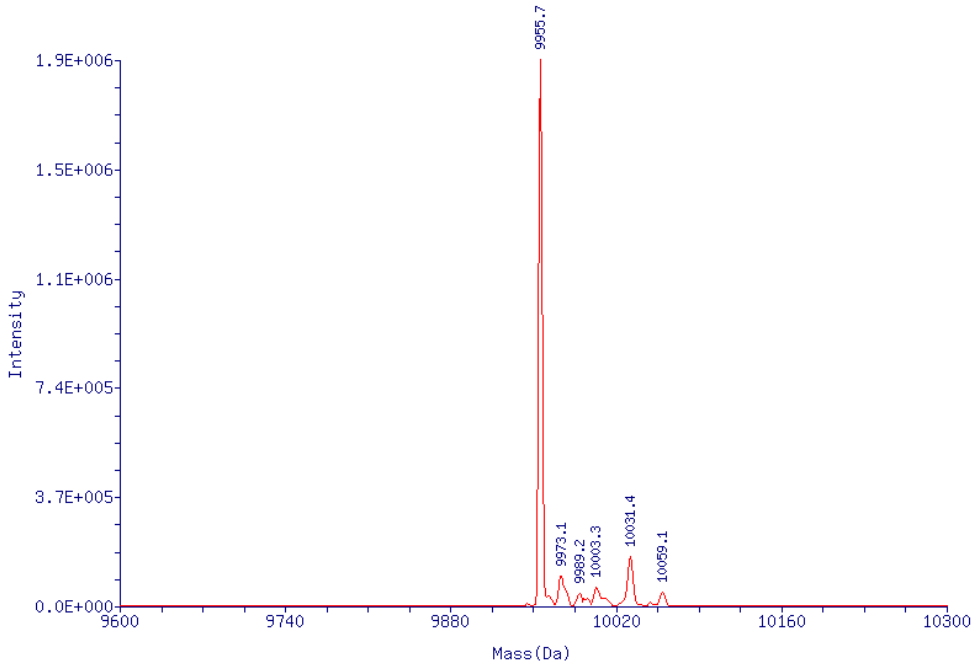


**Compound B09**.

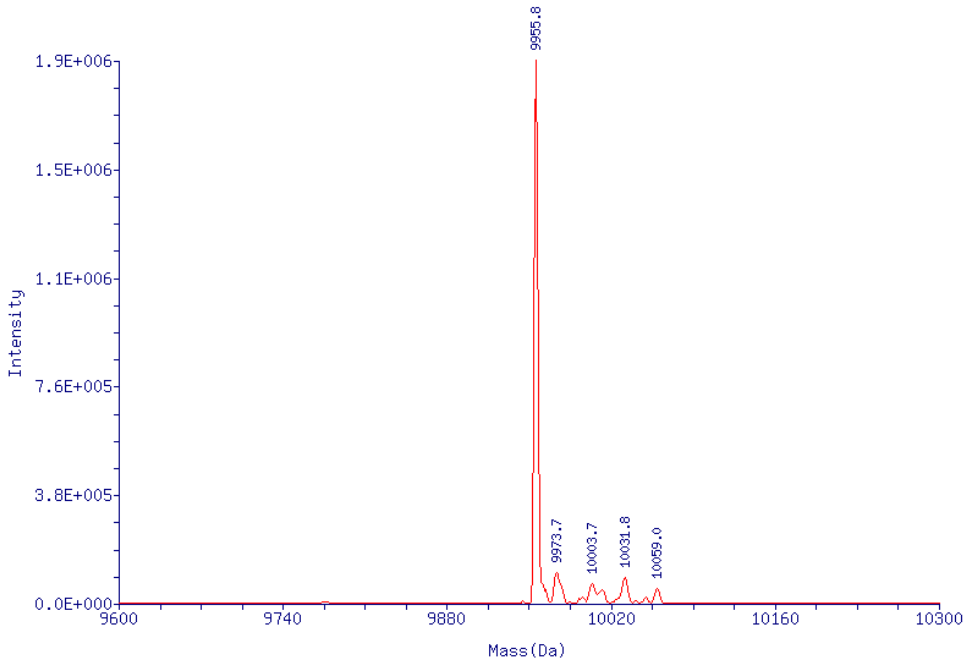


**Compound B10**.

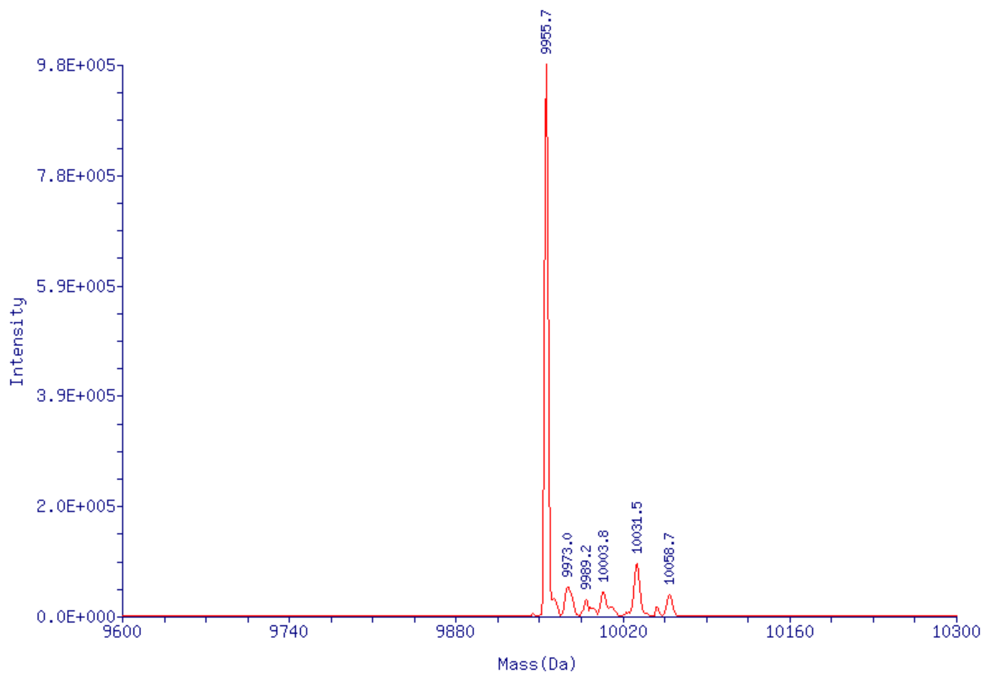


**Compound B11**.

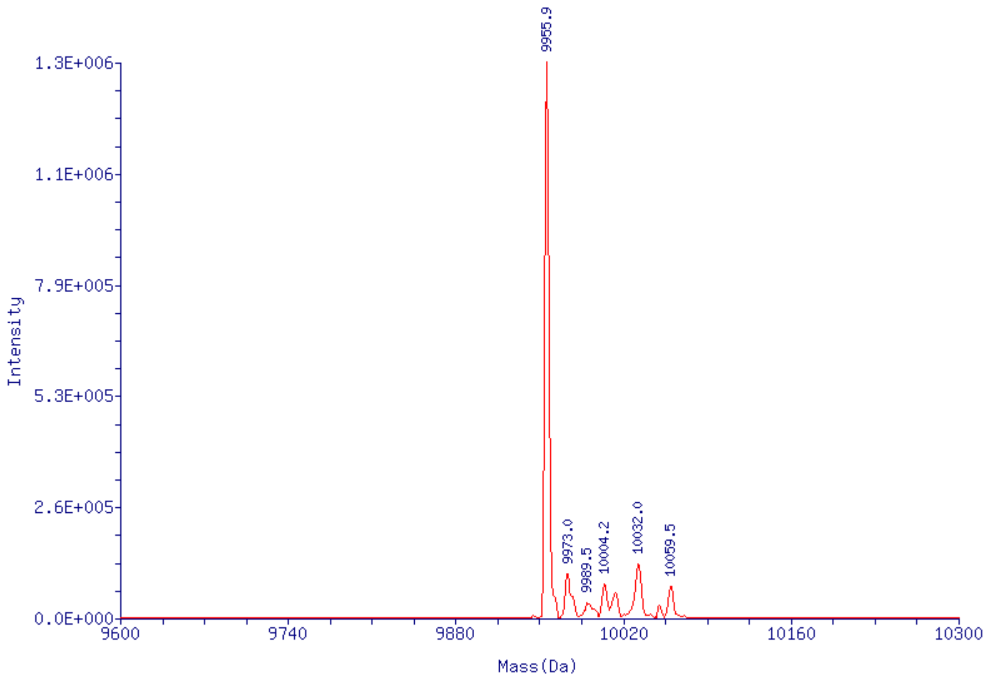


**Compound B12**.

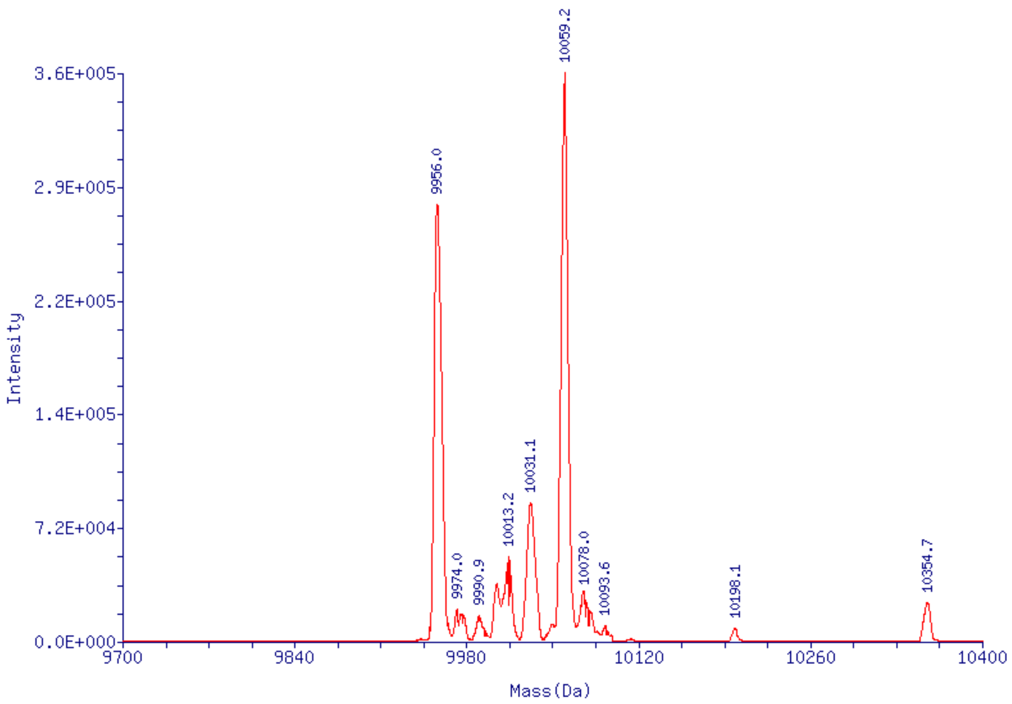


**Compound C01**.

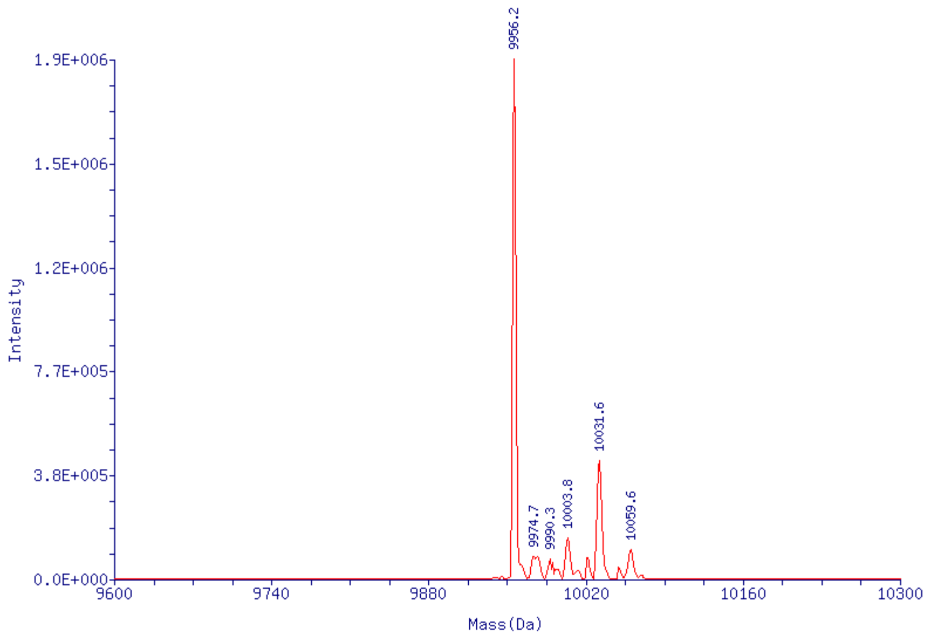


**Compound C02**.

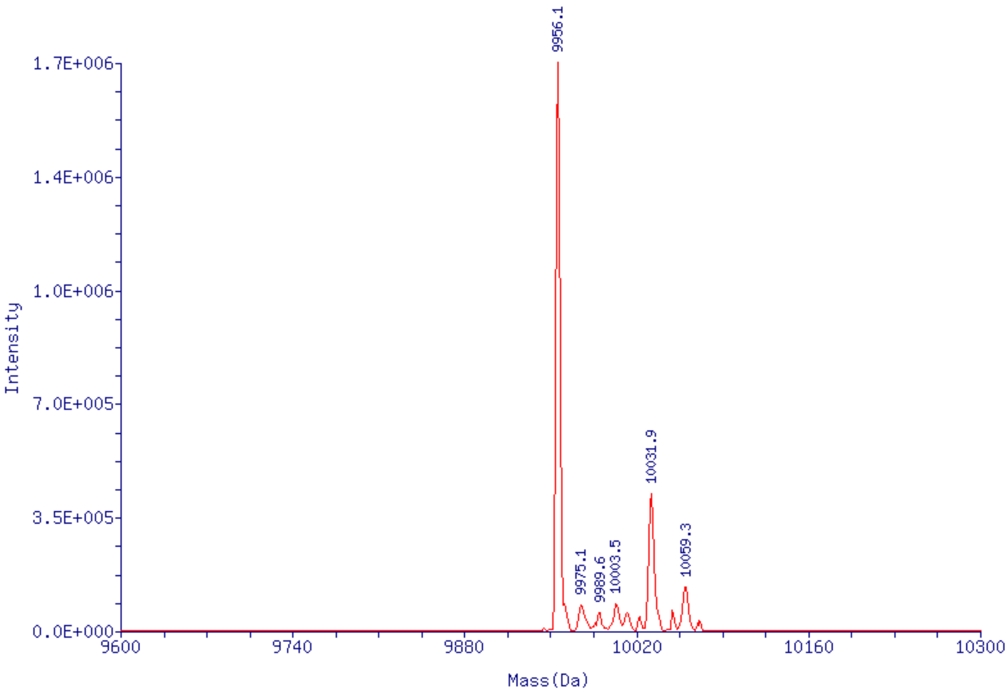


**Compound C03**.

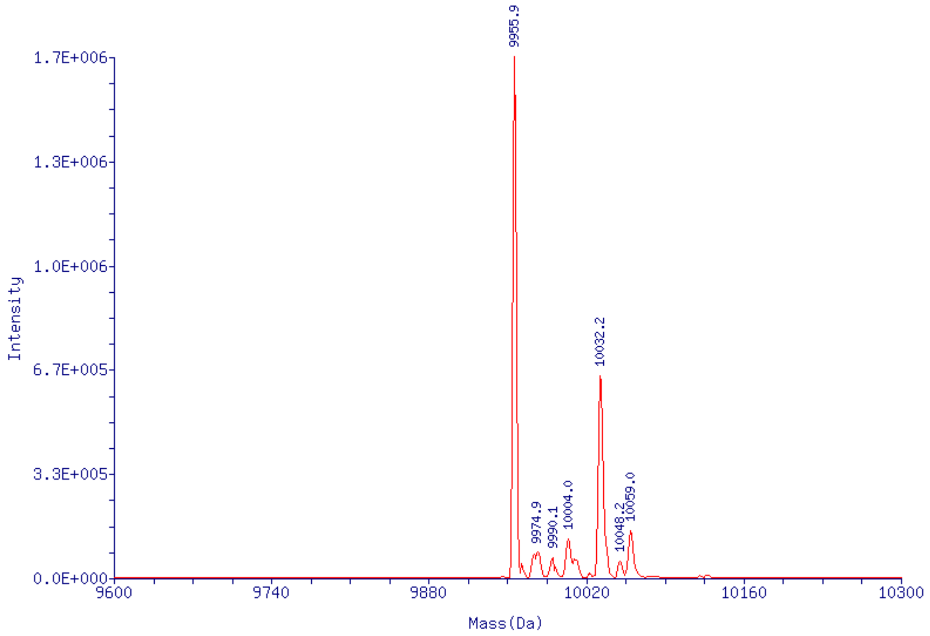


**Compound C04**.

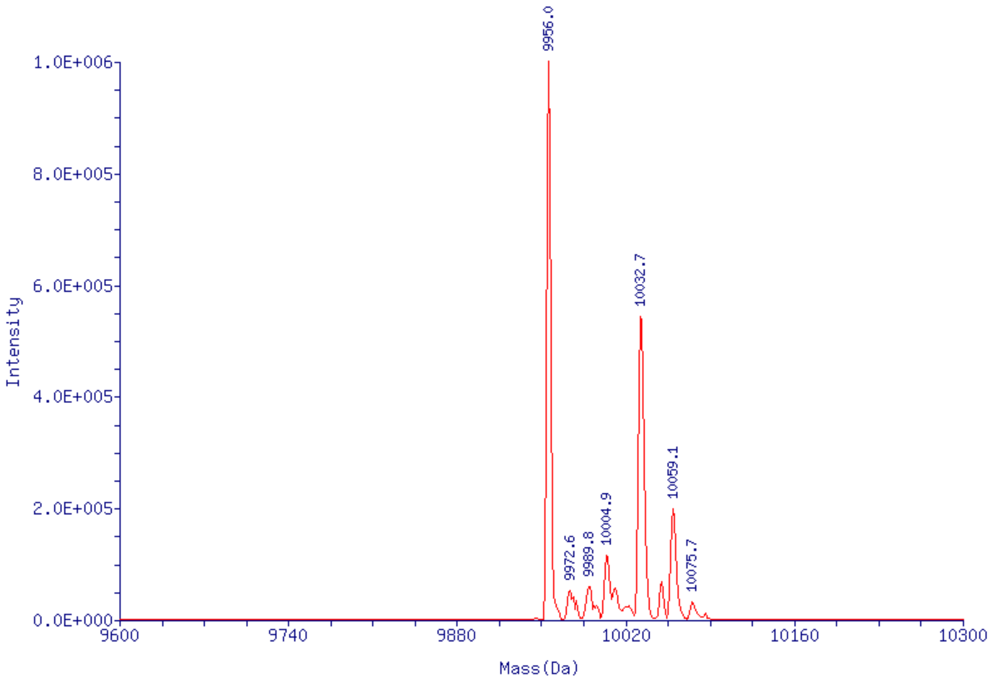


**Compound C05**.

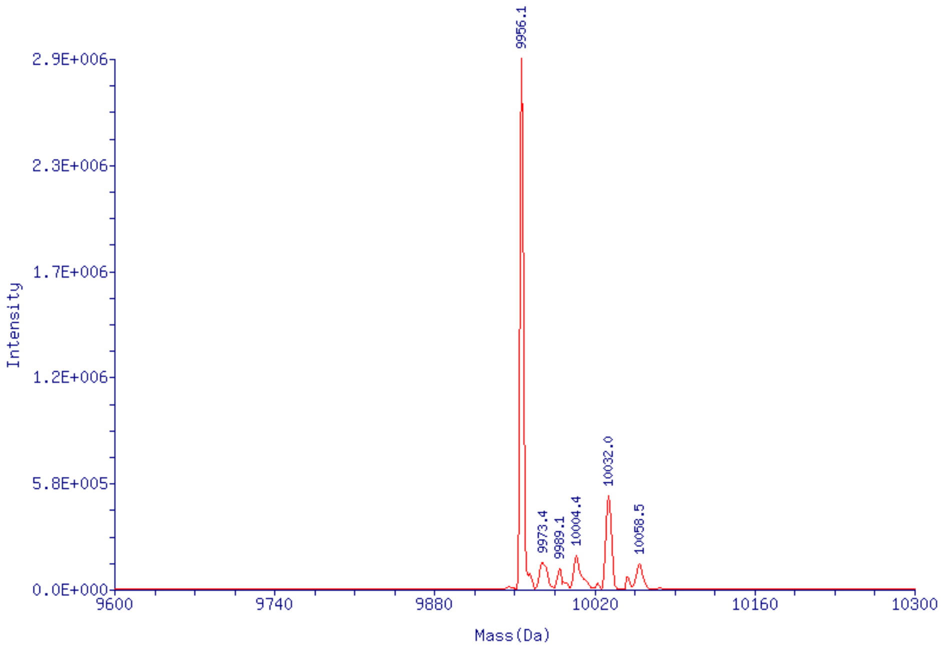


**Compound C06**.

## 8. 16mer Conjugation Data with A04 & A05

*Thiol-Modified 16-nt RNA*

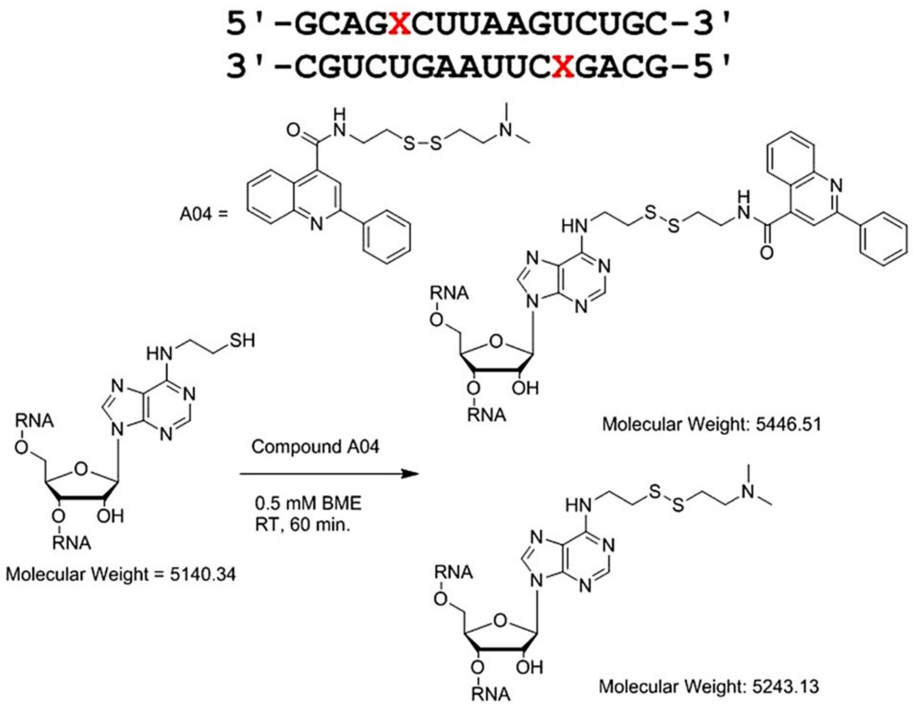


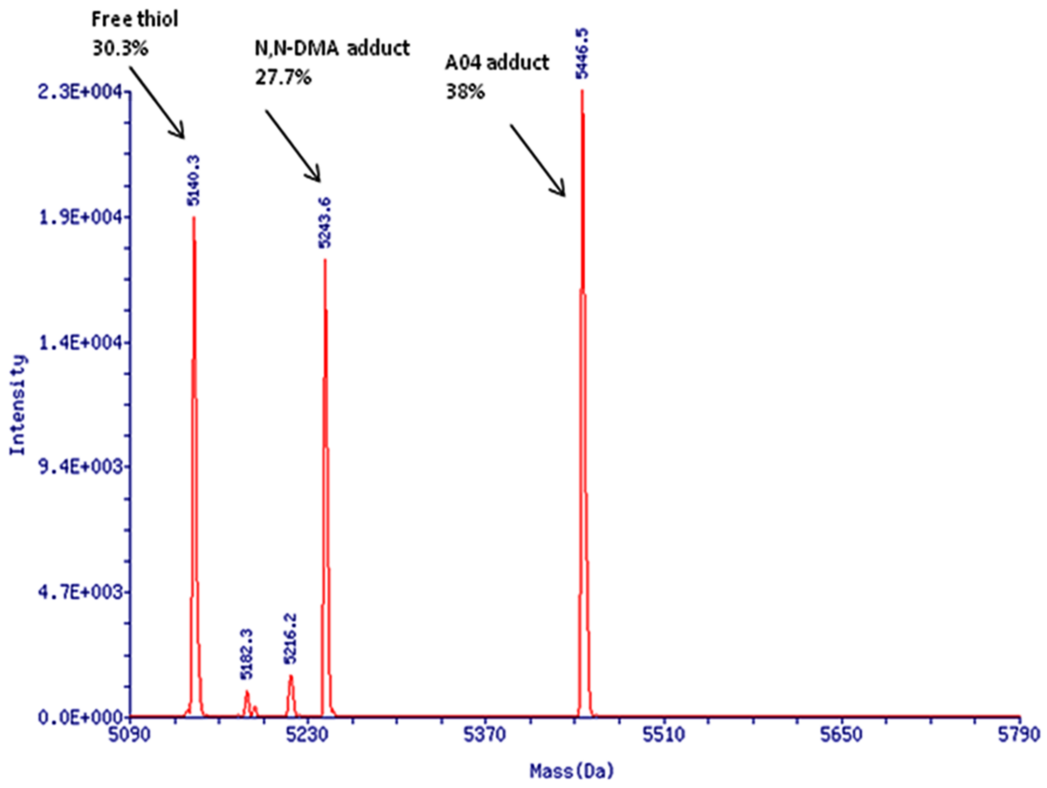

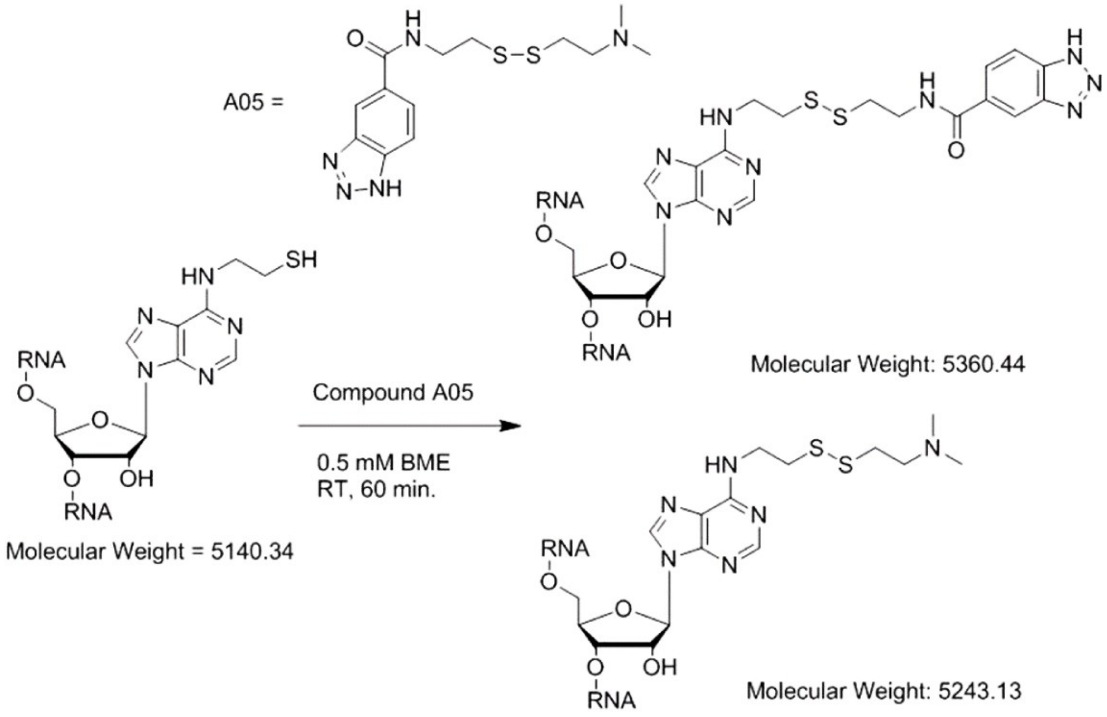
A05 =
Compound A05
0.5 mM BME
RT, 60 min.
Molecular Weight = 5140.34
Molecular Weight: 5360.44
Molecular Weight: 5243.13

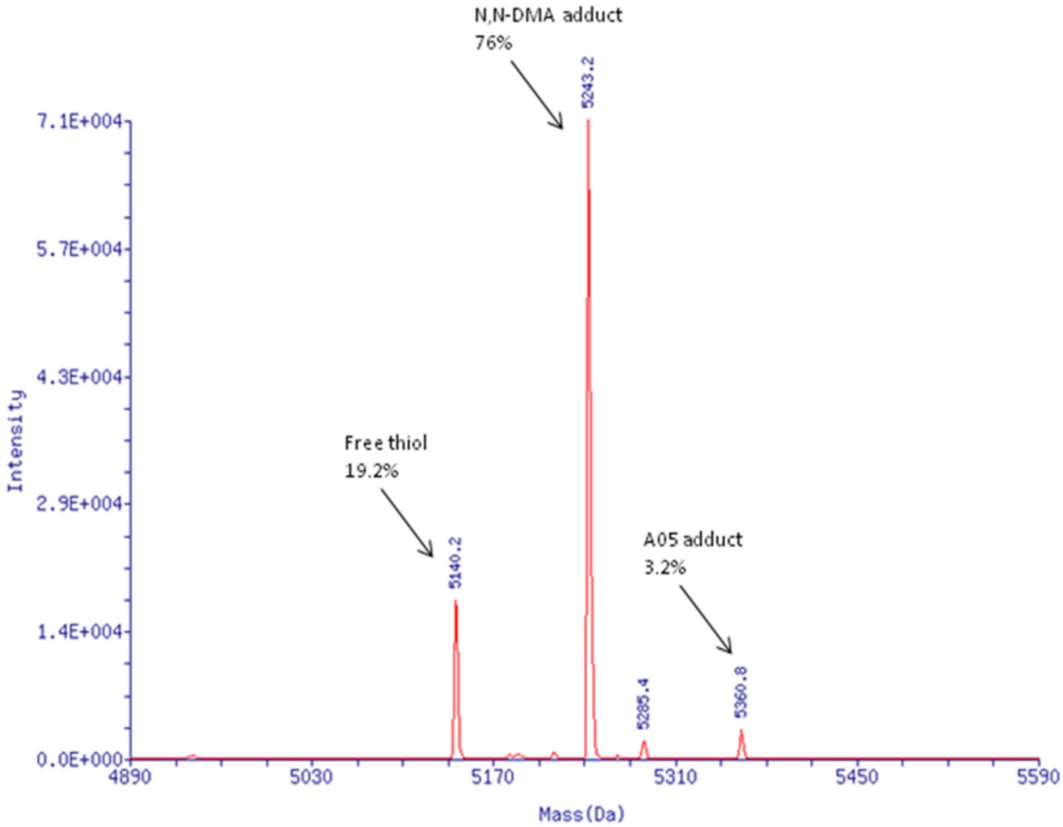
N,N-DMA adduct
76%
Free thiol
19.2%
A05 adduct
3.2%
5243.2
5140.2
5285.4
5360.8
Intensity
Mass (Da)

## 9. Denaturing Gel Electrophoresis of RNA2 after Solid-phase Synthesis

The oxidation conditions used during the solid-phase synthesis of these oligonucleotides are:

- 0.02 M Iodine in pyridine/THF/water
- 15 s per cycle

A 19% polyacrylamide denaturing gel with a xylene cyanol blue standard was used to assess the solid phase RNA synthesis product. Approximately 12 nmol of sample was loaded into the lane and visualized by UV shadowing.

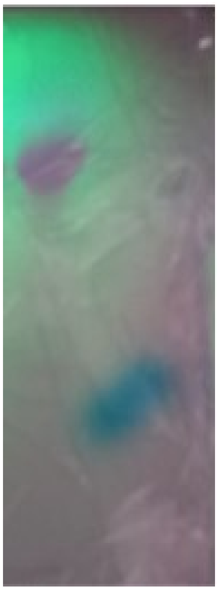

Supplement: Supplementary file 1 [file molecules-20-04148-s001.pdf]
